# Phytochemical Elucidation and Effect of *Maesa indica* (Roxb.) Sweet on Alleviation of Potassium Dichromate-Induced Pulmonary Damage in Rats

**DOI:** 10.3390/plants13030338

**Published:** 2024-01-23

**Authors:** Fatma Alzahra M. Abdelgawad, Seham S. El-Hawary, Essam M. Abd El-Kader, Saad Ali Alshehri, Mohamed Abdelaaty Rabeh, Aliaa E. M. K. El-Mosallamy, Abeer Salama, Rania A. El Gedaily

**Affiliations:** 1 Department of Pharmacognosy, Faculty of Pharmacy, Heliopolis University El Salam City, Cairo 11785, Egypt; fatma.mahrous@hu.edu.eg; 2 Department of Pharmacognosy, Faculty of Pharmacy, Cairo University, Giza 11562, Egypt; seham.elhawary@yahoo.com; 3 Department of Timber Trees Research, Horticultural Research Institute (ARC), Giza 12619, Egypt; eltorifi_ola@yahoo.com; 4 Department of Pharmacognosy, College of Pharmacy, King Khalid University, Abha 62251, Saudi Arabia; salshhri@kku.edu.sa (S.A.A.); mrabeh@kku.edu.sa (M.A.R.); 5 Department of Pharmacology, National Research Centre, Cairo 12622, Egypt; aliaamoneer@hotmail.com (A.E.M.K.E.-M.); berrotec@yahoo.com (A.S.)

**Keywords:** *Maesa indica*, LC/MS, GSH, MDA, AKt, PI3K, rat

## Abstract

*Maesa indica* (Roxb.) Sweet is one of the well-known traditionally-used Indian plants. This plant is rich in secondary metabolites like phenolic acids, flavonoids, alkaloids, glycosides, saponins, and carbohydrates. It contains numerous therapeutically active compounds like palmitic acid, chrysophanol, glyceryl palmitate, stigmasterol, *β*-sitosterol, dodecane, maesaquinone, quercetin 3-rhaminoside, rutin, chlorogenic acid, catechin, quercetin, nitrendipine, 2,3-dihydroxypropyl octadeca-9,12-dienoate, kiritiquinon, and *β*-thujone. The *Maesa indica* plant has been reported to have many biological properties including antidiabetic, anticancer, anti-angiogenic, anti-leishmanial, antioxidant, radical scavenging, antibacterial, antiviral, and anti-coronavirus effects. One purpose of the current study was to investigate the leaves’ metabolome via Triple-Time-of-Flight-Liquid-Chromatography-Mass Spectrometry (T-TOF LC/MS/MS) to identify the chemical constituents of the *Maesa indica* ethanolic extract (ME). Another purpose of this study was to explore the protective effect of ME against potassium dichromate (PD)-induced pulmonary damage in rats. Rats were assigned randomly into four experimental groups. Two different doses of the plant extract, (25 and 50 mg/kg), were administered orally for seven consecutive days before PD instillation injection. Results of our study revealed that ME enhanced cellular redox status as it decreased lipid peroxidation marker, MDA and elevated reduced glutathione (GSH). In addition, ME upregulated the cytoprotective signaling pathway PI3K/AKT. Moreover, ME administration ameliorated histopathological anomalies induced by PD. Several identified metabolites, such as chlorogenic acid, quercetin, apigenin, kaempferol, luteolin, and rutin, had previously indicated lung-protective effects, possibly through an antioxidant effect and inhibition of oxidative stress and inflammatory mediators. In conclusion, our results indicated that ME possesses lung-protective effects, which may be the result of its antioxidant and anti-inflammatory properties.

## 1. Introduction

*Maesa indica* Roxb. Sweet, also known as wild berry or wild tea, is a long, glabrous, evergreen shrub belonging to the family Primulaceae and native to China and Southern India [1]. It can also be found in forests with high humidity, semi-evergreens, and evergreens. Numerous phytochemical classes, such as flavonoids, phenolics, saponins, tannins, carbohydrates, fixed oil, and glycosides, have been identified in this plant [1]. Palmitic acid, chrysophanol, glyceryl palmitate, stigmasterol, β-sitosterol, dodecane, maesaquinone, quercetin 3-rhamnoside, rutin, chlorogenic acid, catechin, quercetin, nitrendipine, 2,3-dihydroxypropyl octadeca-9,12-dienoate, kiritiquinon, and β-thujone are among the many active ingredients identified in it [1,2]. *Maesa indica* is reported to have many biological properties like anticancer, antidiabetic [2], anti-angiogenic, anti-leishmanial, antioxidant, radical scavenging [2], antibacterial [3], antiviral [4] and anti-coronavirus [5].

The LC-MS method is a strong analytical tool for profiling plant metabolites. Plants biochemistry is moderately complex and includes several semi-polar components, including important secondary metabolite classes, which may be effectively separated and identified using LC-MS techniques [6]. Electrospray ionization (ESI), both in positive and negative ion modes, has quickly become the method of choice for secondary metabolites analysis, particularly when used in conjunction with MS-MS techniques. The method makes it possible to analyze pure and mixed samples quickly and without the requirement for derivatization by using small amounts of ingredients (less than 1 mg).

Natural polyphenols are a broad class of compounds present in plant-based food that offer protection against neurological, metabolic, cardiovascular, and cancerous diseases [7]. Due to well-established evidence of their antioxidant activities both in vitro and in-vivo, polyphenols have garnered significant interest as potential therapeutic agents in lung degenerative disorders, particularly in acute lung injury [7,8].

Acute lung injury (ALI), also known as acute respiratory distress syndrome (ARDS), is a severe form of acute inflammatory lung injury linked to increased morbidity and mortality as well as the development of multiple organ failure [8]. ALI affects more than 3 million patients a year, accounting for up to 10% of intensive care unit (ICU) patients [9]. Diffuse alveolar epithelial destruction, pulmonary vascular endothelial cells, neutrophil infiltration, and the flow of protein-rich fluid into the alveolar gaps are among the primary pathological characteristics of acute lung injury. Inflammatory responses, apoptosis, redox imbalance, and goblet cell hyperplasia (GCH) are a few possible ALI causes [10]. 

Large amounts of hazardous chromium compounds are released into the environment as a result of the widespread usage of chromium in industry. While the hexavalent form of chromium is typically toxic, the trivalent form of chromium is less toxic and soluble [11]. Reactive oxygen species (ROS) are produced when hexavalent chromium is reduced, and these ROS have the potential to harm DNA, lipids, and proteins in cells [12]. In both experimental and clinical studies, the involvement of oxidants and oxidative damage in the etiology of ALI/ARDS has been extensively described [12]. Oxidative stress can result from an imbalance in the production and elimination of ROS by the antioxidant defense system [10]. Many inflammatory diseases, including idiopathic pulmonary fibrosis (IPF), acute respiratory distress syndrome, cystic fibrosis, and human immunodeficiency diseases, have been linked to changes in glutathione (GSH) and malondialdehyde (MDA) levels [13]. According to [14], the phosphatidylinositol-3-kinase (PI3K)/protein kinase B (AKT) is thought to be connected to cell division, death, metabolism, and proliferation. Many recent research investigations have focused on the relationship between PI3K/AKT and pulmonary protection as it is believed that PI3K/Akt promotes the survival of pulmonary cells [15]. 

The objective of this study was to investigate the lung-protective effect of ME in potassium dichromate-induced pulmonary injury through measuring the levels of GSH, MDA, AKt, and PI3K as well as exploring the prevention of histopathological changes associated with exposure to PD. In addition, the chemical profile of ME was analyzed using Triple-Time-of-Flight-Liquid-Chromatography-Mass Spectrometry (T-TOF LC/MS/MS) to identify constituents that may be relevant to the plant’s pulmonary protective action.

## 2. Results and Discussion

### 2.1. Metabolic Profiling Using T-TOF LC/MS/MS

**Figure 1:**
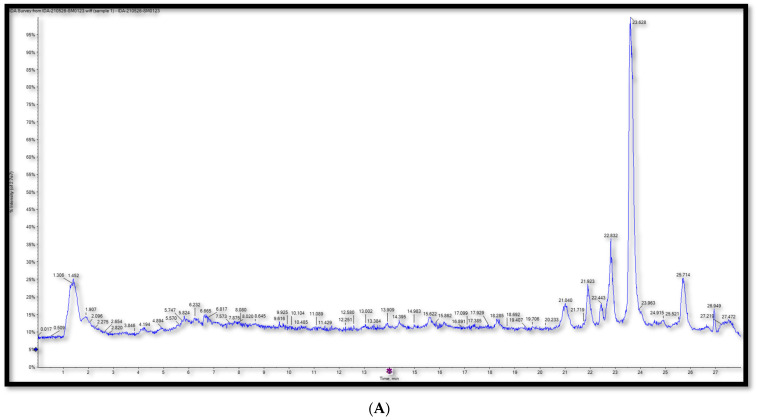

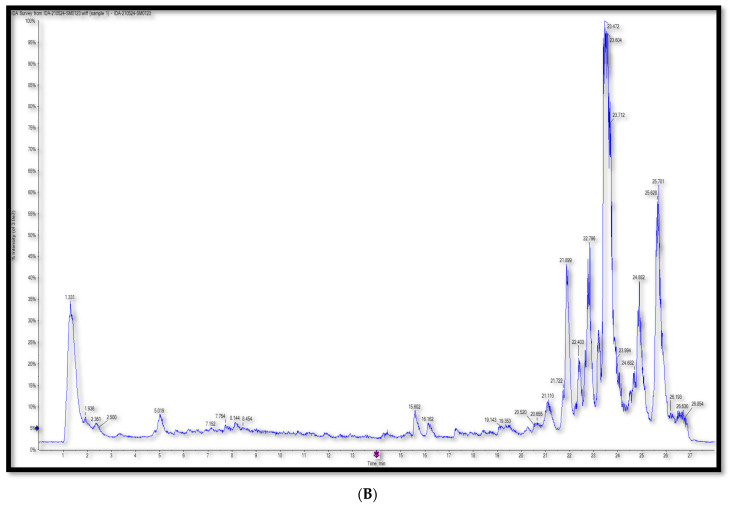
UPLC-ESI-TOF-MS base peak chromatograms of 70% ethanol extract of *Maesa indica* (**A**), (−): negative ESI mode and (**B**), (+): positive ESI mode.

In this study, we sought to provide a metabolite profile of ME using UPLC–ESI–TOF-MS and correlate the expected anti-pulmonary damage activity to its phytochemical composition. Fifty-six metabolites were detected in ME. The metabolites were classified according to their chemical structure. We found 26 flavonoids, 17 amino acids, 8 phenolics, 3 sugars, and 2 alkaloids. For flavonoid identification, differentiating between O and C-linked flavonoid and phenolic conjugates required tandem mass-spectrometric analyses (MS^2^). While the fragmentation pattern of the C-glycosyl conjugates shows major fragment ions as a result of neutral losses of 90 and 120 amu of pentose and hexose sugars, respectively, the *O*-glycosyl attachment typically can be easily identified in MS^2^ spectra by the neutral losses of 162, 146, and 132 amu indicative of hexose, deoxyhexose, or pentose moieties, respectively [16]. The base peak chromatograms of ME in the negative and positive ESI modes are depicted in Figure 1. The details of the detected and assigned major metabolites are listed in Table 1.

#### 2.1.1. Flavonoids

A total of 26 flavonoid derivatives were detected in the 70% ethanol metabolite profile of *Maesa indica.* The identified flavonoids were 13 flavonols, 9 flavones, 3 flavanones, and 1 flavanol. The peak identifications depended on the unique fragmentation pattern of each compound as losses of small molecules from the [M + H]^+^ ion or [M − H]^−^. For example, a loss of 18, 28, or 42 amu indicates the loss of (H_2_O), (CO), (CH_2_CO), respectively [17]. 

##### Flavonol Derivatives

Thirteen flavonol derivatives were identified in ME metabolite profile. The common fragmentation of flavonols starts with the dehydration of product ions [M + H − H_2_O]^+^, followed by sequential losses of CO [M + H − H_2_O − CO]^+^ and [M − H − H_2_O − 2CO]^+^. Myricetin was detected in Peak No. 3 with molecular ion *m*/*z* 317.0546 [M − H]^−^ (t_r_ = 1.2215 min, [C_15_H_9_O_8_]^−^), which produces distinctive fragments at *m*/*z* 281.1009 due to dehydration [M − H − 2H_2_O]^−^ and *m*/*z* 225.0102 [M − H − 2H_2_O − 2CO]^−^ [16] (Figure S1). Quercetin-3-Glucuronide (t_r_ = 1.338 min, [C_12_H_17_O_13_]^−^) with the molecular ion *m*/*z* 477.0106 [M − H]^−^ and Quercetin-3-D-xyloside (t_r_ = 1.415 min, [C_20_H_17_O_11_]^−^) with the molecular ion *m*/*z* 477.0106 [M − H]^−^ were identified at Peaks 7 and 10, respectively. The specific MS2 fragment at 301.0204 corresponds to the aglycone quercetin moiety after the loss of glucuronic acid molecule and the loss of xylose moiety [18,19] (Figures S2 and S3). Peak No. 29 was identified as kaempferol 7-neohesperidoside with the molecular ion *m*/*z* 593.1576 [M − H]^−^ (t_r_ = 6.308 min, [C_27_H_29_O_15_]^−^). We observed an MS2 daughter peak at *m*/*z* 285.0364 for the kaempferol moiety [20] (Figure S4). Peak 30, which appeared at t_r_ = 4.908 min, was interpreted as rutin (Quercetin-*O*-rutinoside), with the molecular ion *m*/*z* 611.16034 [C_27_H_31_O_16_]^+^ showing the characteristic fragment of [M + H − 308]^+^ at *m*/*z* 303.110, attributed to the loss of glucose and rhamnose moieties [21] (Figure S5). Peak No. 34 was tentatively identified as 3′-methoxy-4′,5,7-trihydroxyflavonol, with the molecular ion *m*/*z* 315.1088 (t_r_ = 5.3938 min, [C_16_H_11_O_7_]^−^) with a fragment peak at *m*/*z* 300.236 [M − H − CH3]^−^ [22]. Peak No. 36 was identified as Quercetin-4′-glucoside (t_r_ = 6.1606 min, [C_21_H_19_O_12_]^−^), with the molecular ion *m*/*z* 463.0924 [M − H]^−^ and a specific daughter peak at *m*/*z* 301.0264 [(M − H) − glucose]^−^ [6] (Figure S6). Quercitrin (Quercetin−3−O−rhamnoside) was ascribed to molecular ion *m*/*z* 447.0976 [M − H]^−^ (Peak 37, t_r_ = 6.224 min, [C_21_H_19_O_11_]^−^). The aglycone peak at *m*/*z* 301.11 was caused by the loss of rhamnose [(M − H) − rhamnose] [23]. Peak No. 41 was interpreted as Kaempferol-3-*O*-glucoside with the molecular ion *m*/*z* 447.0948 [M − H]^−^ (t_r_ = 6.6201 min, [C_21_H_19_O_12_]^−^) and a specific fragment at *m*/*z* 285.0409 [M − H − 162]^+^, which resulted from the loss of a glucose moiety, which in turn left a remainder of kaempferol aglycone [24] (Figure S7). Isorhamnetin-3-*O*-glucoside was recognized at Peak 42 with the molecular ion *m*/*z* 477.0996 [M − H]^−^ (t_r_ = 6.734 min, [C_22_H_21_O_12_]^−^). We identified remarkable fragments at *m*/*z* 315.2062 due to the loss of the glucose moiety [M − H − glucose]^−^ and *m*/*z* 243.2670 due to dehydration [M − H − glucose − H_2_O]^−^ [25] (Figure S8). Quercetin was detected in both the positive and negative mode. In the positive mode, it was annotated to the molecular ion at *m*/*z* 303.0460 [M + H]^+^ (peak 43, t_r_ = 7.078 min, [C_15_H_11_O_7_]^+^) and the observed daughter peak in MS2 spectra at *m*/*z* [285.1349] ^+^, implying the neutral loss of 18 amu for water moiety. There were two daughter peaks in MS2 spectra at *m*/*z* [153.2648 and 137.0592] ^+,^ representing ring B and ring A of flavonol, respectively [17] (Figure S9). Peak number 44 (t_r_ = 7.093 min, [C_21_H_21_O_12_]^+^) in the positive mode was interpreted as hyperoside (quercetin-3-*O*-galactoside). Its molecular ion was *m*/*z* 465.1005 [M + H]^+^ (t_r_ = 7.093 min, [C_21_H_21_O_12_]^+^), with characteristic fragment ions at *m*/*z* 303.0500 [(M + H)-galactose] [19] (Figure S10). At t_r_ = 7.75 min, Peak No. 46 was recognized as 3, 5, 7-trihydroxy-4′-methoxyflavone (Diosmetin), with the molecular ion *m*/*z* 301.0998 [C_16_H_13_O_6_]^+^ having a dehydration peak at 283.0971 after loss of H_2_O, followed by another characteristic peak at 255.0724 due to loss of 28 amu representing the loss of CO [26] (Figure S11). 

##### Flavone Derivatives

There were nine identified flavone compounds in the ME chemical profiling. The first one was Kaempferol-3-*O*-alpha-L-arabinoside at peak 12 with the molecular ion *m*/*z* 417.0578 [M − H]^−^ (t_r_ = 1.441 min, [C_20_H_17_O_10_]^−^), and MS2 peaks at *m*/*z* 227.0349 for [M − H − 2HCO]^−^ and *m*/*z* 285.0406 for the kaempferol moiety [27] (Figure S12). Peak No. 35 was interpreted as baicalein-7-*O*-glucuronide (tr = 5.7484 min, [C_21_H_17_O_11_]^−^), with the molecular ion *m*/*z* 445.135 [M − H]^−^ and a pattern of fragmentation at *m*/*z* 269.0450 [M − H − 176], corresponding to the loss of glucuronic acid [28] (Figure S13). Acacetin-*O*-rutinoside (linarin) was assigned to the molecular ion *m*/*z* 591.1363 [M − H]^−^ at peak 38 (t_r_ = 6.237 min, [C_28_H_31_O_14_]^−^), and its fragmentation yielded MS2 fragment peaks at *m*/*z* 445.101, representing the loss of deoxyhexose [M − H − rhamnose]^−^ and *m*/*z* 283.0327, representing the aglycone moiety [29] (Figure S14). Peak No. 39 was identified as luteolin (t_r_ = 6.237 min, [C_15_H_9_O_6_]^−^), with the molecular ion *m*/*z* 285.0396 [M − H]^−^ and a pattern of fragmentation at *m*/*z* 163.6529 and *m*/*z* 151.005, corresponding to ^1,3^A^−^ and ^1,3^B^−^ anions, respectively, which formed via the 1,3-retrocyclization cleavage of the ionized luteolin molecule [17,30] (Figure S15). The molecular ions and the aglycone fragment ions of apigenin-7-*O*-glucoside were detected at *m*/*z* 431.0983 and 269.00423 [M − H − glucose]^−^, respectively, at Peak 47 (t_r_ = 7.831 min, [C_21_H_19_O_10_]^−^) [28] (Figure S16). Formononetin was detected at Peak No. 51 (t_r_ = 8.8604 min, [C_16_H_13_O_4_]^+^) with the molecular ion *m*/*z* 269.1138 [M + H]^+^ and MS2 fragment peaks at *m*/*z* 254.0577 and 213.0736, corresponding to the loss of a methyl group [M + H − CH_3_]^+^ and two carbon monoxide molecules [M + H − 2CO]^+^, respectively [6] (Figure S17). Peak No. 52 was identified as apigenin (t_r_ = 10.0624 min, [C_15_H_9_O_5_]^−^), with the molecular ion *m*/*z* 269.0419 [M − H]^−^ and a specific MS2 fragment peak at *m*/*z* 117.0327, corresponding to [M + H − 2H_2_O − 2CO_2_ − CO]^+^ [31] (Figure S18). The final identified flavone was apigenin 8-C-glucoside (vitexin), at Peak 54 (t_r_ = 12.3865 min, [C_21_H_21_O_10_]^+^), with the molecular ion *m*/*z* 433.114 [M + H]^+^ and specific MS2 fragment peaks at *m*/*z* 415.1061, corresponding to the dehydration [M + H − H_2_O]^+^, and at *m*/*z* 313.0744 [(M + H) − 120], which is characteristic to the C-glycosyl conjugates [6] (Figure S19).

##### Flavanone Derivatives

Three flavanones were identified in the extract, three in the negative mode and one in the positive mode. Peak No. 1 was attributed to naringenin (tr = 1.1824 min, [C_15_H_11_O_5_]^−^), with the molecular ion *m*/*z* 271.0123 [M − H]^−^ and a fragmentation pattern at *m*/*z* 151.005, 119.051 and 107.014 [32] (Figure S20). The second flavanone was hesperetin, at Peak No. 45 (tr = 7.4872 min, [C_16_H_13_O_6_]^−^) with molecular ion *m*/*z* 301.1187 [M − H]^−^ and specific MS2 peaks at *m*/*z* 283.1092 and 268.0583, attributed to the loss of water [M − H − H_2_O]^−^ and the loss of methyl group [M − H − H_2_O − CH_3_]^−^, respectively [16] (Figure S21). The third was 3′ 4′ 5 7-tetrahydroxyflavanone (fustin), found at Peak No. 53 (tr = 11.3895 min, [C_15_H_13_O_6_]^+^), with the molecular ion *m*/*z* 289.1188 [M + H]^+^ and a specific fragmentation pattern at *m*/*z* 179.0339, 163.039, and 153.0184 [33] Figure S22.

##### Flavanol Derivatives

One flavanol was identified in the ME analysis. This flavanol was catechin at Peak No. 34 (tr = 5.535 min, [C_15_H_13_O_6_]^−^), with the molecular ion *m*/*z* 289.011 [M − H]^−^ and a specific MS2 peak at *m*/*z* 245.098 [34] (Figure S23).

##### Phenolic Acids 

Phenolic acids are usually found in plant extracts. These secondary metabolites have a variety of biological properties, such as anti-inflammatory, hepato-protective, antioxidant, antibacterial, cardioprotective, antidiabetic, anticancer, lung-protective, and neuroprotective properties [35]. Eight peaks were tentatively identified as phenolic acids, specifically chlorogenic acid, *trans*-cinnamic acid, *p*-hydroxybenzoic acid, protocatechuic acid, rosmarinic acid, γ-tocotrienol, daphnetin, and caffeic acid. These eight phenolic acids were assigned to Peaks 19, 24, 26, 31, 48, 49, 50, and 55, respectively. They are represented in Figures S24–S29, respectively. Chlorogenic acid had the molecular ion peak [M − H]^−^ at *m*/*z* 353.0888 and MS2 fragment ion peaks at *m*/*z* 191.0546, corresponding to [C_7_H_11_O_6_]^−^ residue and *m*/*z* 173.0496 for additional dehydration [C_7_H_11_O_6_ − H_2_O]^−^ [36]. *P*-hydroxybenzoic acid (tr = 3.2552 min, *m*/*z* 137.0248, [C_7_H_5_O_3_]^−^), protocatechuic acid (tr = 4.9513 min, *m*/*z* 153.0183 [C_7_H_5_O_4_]^−^), daphnetin (tr = 8.054 min, *m*/*z* 179.1066, [C_9_H_7_O_4_]^+^), and caffeic acid (tr = 14.1078 min, *m*/*z* 179.0549, *m*/*z* 179.0549, [C_9_H_7_O_4_]^−^) all have specific MS2 peaks due to the loss of CO2 [M − H − CO_2_]^−^ at 93.0341, 109.0297, and 135.0644, respectively [37]. Rosmarinic acid (tr = 7.8473, min, [C_18_H_15_O_8_]^−^) with the molecular ion *m*/*z* 359.0178, has a characteristic MS2 daughter peak at 161.0540 [M − H − C_9_H_9_O5 − 2H_2_O]^−^ [38]. *Trans*-cinnamic acid (tr = 2.3538 min, [C_9_H_8_O_2_]^+^), with the molecular ion *m*/*z* 149.0598, was identified with specific MS2 peaks at 121.0281 and 65.0367 [16]. Peak No. 49 corresponded to γ-tocotrienol (vitamin E), with the molecular ion *m*/*z* 411.1733 and characteristic daughter peaks at 409.8878 and 242.12191 [6].

##### Alkaloids

Alkaloids are very strong therapeutic compounds, and they have many reported biological properties including antimitotic, anticancer, anti-inflammatory, analgesic, antibacterial, antifungal, local analgesic, pain-relieving, antioxidant, antiparasitic, antiplasmodic, antibacterial, anti-HIV, and local anesthetic capabilities [39]. Two alkaloids, trigonelline and caffeine, were tentatively identified in our chemical study of ME. Peak No. 11 was assigned to trigonelline (tr = 1.4326, min, [C_7_H_8_NO_2_]^+^), with the molecular ion 138.0522 and a fragment ion at *m*/*z* 92.0489 indicating the loss of an ethyl unit [M + H − C2H6O]^+^ [40] (Figure S20). Caffeine (tr = 5.0098, min, [C_8_H_11_N_4_O_2_]^+^) was assigned to Peak No. 32, with the molecular ion *m*/*z* 195.0863 and an MS2 peak at *m*/*z* 138.052 corresponding to [M +H − OCNCH_3_]^+^ [6] (Figure S31).

Many compounds could only be identified in only one ionization mode during the LC-MS analysis, because the chemical stability of these compounds is higher in one mode. We used both the negative and the positive ionization LC-MS mode to enhance the chemical profiling of the plant extract by studying the fragmentation pathway in the negative and the positive ion modes. The study confirmed the presence of phenolic acids, flavonoids, alkaloids, nitrogenous compounds, amino acids, and carbohydrates. The major classes were flavonoids and phenolics, which are characterized by having many therapeutic effects, such as anti-inflammatory, hepatoprotective, antioxidant, antiaging, lung protection, cardioprotective, antidiabetic, anxiolytic, neuroprotective, immunomodulatory, gastroprotective, hormone synthesis regulation, and anti-Alzheimer’s [41,42].

To our knowledge, only a few studies have been done to study the chemical composition of the *Maesa indica* plant. This was the first screening and analysis of ME using LC/MS/MS, and it resulted in the identification of fifty-six compounds. Only five of the identified compounds were reported to be present in ME in previous studies. The previously reported compounds were chlorogenic acid, quercetin, quercetrin, catechin, and rutin. The remaining fifty-one compounds were identified for the first time in this study.

### 2.2. In-Vivo Study

#### 2.2.1. Acute Toxicity

Male and female mice treated with a single dose of 0.5 g/kg of ME did not exhibit any toxicity signs such as death, hair loss, gastrointestinal disturbances, yellow spots, or abnormal behavior. As a result, the acute fatal toxicity test confirmed that *Measa indica* extract could be administered orally without a significant risk.

#### 2.2.2. Investigation of the Lung Protection Activity of ME

##### Effect of ME on GSH and MDA Lung Contents 

**Figure 2:**
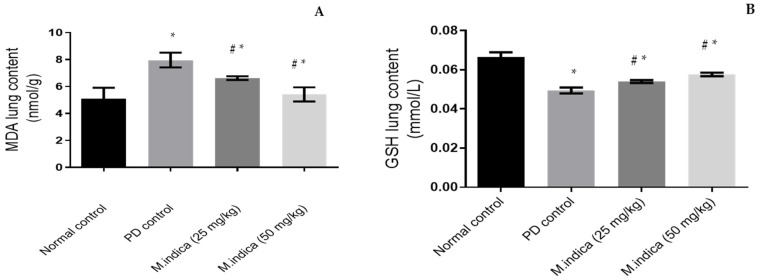
Effect of ME on the lung content of (**A**) GSH in mmol/L. (**B**) MDA in nmol/g. Data were expressed as mean ± SD. Statistical analysis was carried out by one-way ANOVA followed by Tukey’s multiple comparisons test. * Significant difference at *p* < 0.05 when compared to the normal group. ^#^ Significant difference at *p* < 0.05 when compared to the PD group.

PD, containing hexavalent chromium, generates ROS inducing acute lung injury [43]. In the current study, PD instillation significantly (*p*-value < 0.05) reduced GSH lung tissue concentration from 0.0666 ± 0.0023 mmol/L (in the control group) to 0.0494 ± 0.00152 mmol/L (Figure 2A), which means that PD instillation induced a decrease of GSH levels of 26%. PD instillation also significantly (*p*-value < 0.05) increased MDA lung tissue concentration from 5.07771 ± 0.8338 322 nmol/g to 7.9637 ± 0.54515 nmol/g, compared to the control group (Figure 2B), which means that PD instillation induced an increase of MDA levels of 57%.

Interestingly, administration of ME 25 mg/kg significantly (*p*-value < 0.05) increased GSH by 9% and decreased MDA contents by 17% compared to the PD group. As shown in Figure 2, the levels of GSH increased to 0.054 ± 0.00071 mmol/L (vs. 0.0494 ± 0.00152 mmol/L) (A), and MDA contents were decreased to 6.6248 ± 0.140640 nmol/L (vs. 7.9637 ± 0.54515 nmol/g) (B). More effective results were achieved with the high dose of the extract (50 mg/kg). We measured a GSH increase of 16% and an MDA decrease of 32%. Specifically, GSH measured 0.0576 ± 0.00089 mmol/L and MDS measured 5.4268 ± 0.5308 325 nmol/L (Figure 2A and Figure 2B, respectively).

These results indicated that ME, via its antioxidant effect, significantly decreased lung injury caused by the administration of PD. Phenolics scavenge initial free radicals such as hydroxyl radicals and bind metal ion catalysts, decomposing primary oxidation products into non-radical species [44,45]. Phenolic compounds have antioxidant potential; they work by elevating GSH-PX and SOD enzyme levels and decreasing MDA radical levels, which attenuates brain injury [46]. 

In another study, *Maesa indica*, rich in polyphenols, proved to be a radical scavenger with antioxidant properties [2]. *Maesa indica* contains the naturally bioactive compound quercetin, which reduces oxidative damage and chronic diseases such as diabetes, cardiovascular diseases, and hepatotoxicity by reducing amounts of the inflammatory cytokines TNF-α and NF-κ B [47,48]. In addition, *Maesa indica* is a favorite plant of practitioners of folk medicine for its ability to inhibit oxidative hemolysis and its nitric oxide radical and DPPH radical scavenging effects [49]. The highly potent antioxidant compounds quercetin and gallic acid, which were previously identified in ME, have been reported to enhance lung SOD activity and GSH levels and decrease NO and IL-6 levels [50]. On the other hand, the phenolic compounds of *M. lanceolate*, another species of genus *Maesa,* were found to be responsible for its excellent antioxidant activity [51].

##### Effect of ME on AKt and PI3K Lung Contents 

**Figure 3:**
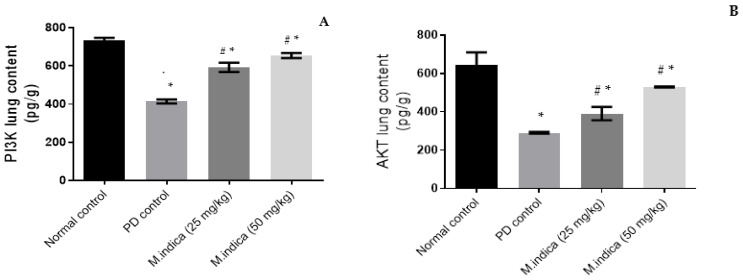
Effect of ME on: (**A**) Lung content of PI3K in pg/g. (**B**) Lung content of AKt in pg/g. Data were expressed as mean ± SD. Statistical analysis was carried out by one-way ANOVA followed by Tukey’s multiple comparisons test. * Significant difference at *p* < 0.05 when compared to the normal group. ^#^ Significant difference at *p* < 0.05 when compared to the PD group.

Intracellular phosphatidylinositol-3 kinase (PI3K) regulates oxidative damage through phosphorylation of AKt, which is a serine/threonine kinase that controls cell survival and stimulates Nrf2 gene expression and nuclear translocation, leading to the inhibition of oxidative stress and inflammation [10]. Our results indicated that the concentration of PI3K in lung tissue was significantly reduced (*p*-value < 0.05) in the PD group as compared to the control group. The concentration fell from 732.5 ± 14.773 pg/g to 413.9 ± 10.2311 pg/g (Figure 3A). This indicates that PD instillation induces a decrease of PI3K lung level with 43%. PD instillation also significantly decreased AKt lung contents (*p*-value < 0.05) in the PD group as compared to the control group. The levels fell from 644 ± 65.761 pg/g to 290.13 ± 4.652 pg/g (Figure 3B), which indicates that PD instillation induces a 55% decrease in AKt levels.

However, administration of ME 25 mg/kg significantly (*p*-value < 0.05) elevated AKt by 35% and PI3K contents by 43% compared to the PD group. As shown in Figure 3, the level of AKt increased to 390.53 ± 34.857 pg/g (vs. 290.13 ± 4.652 pg/g) (A) and PI3K contents increased to 592.2 ± 24.191 pg/g (vs. 413.9 ± 10.2311 pg/g) (B). Larger effects were achieved with the higher dose of the plant extract (50 mg/kg). We measured an AKt elevation of 82% and a PI3K elevation of 58%. Particularly, 529.067 ± 2.891 pg/g were measured for AKt and 654.5 ± 13.233 pg/g for PI3K (Figure 3A,B).

These results strongly suggest that ME possesses antioxidant and anti-inflammatory properties. In line with our results, a previous study showed that phytochemicals of *Maesa indica* have anti-arthritic properties in vitro [52]. In addition, an extract of *Maesa mesozygia*, another species of genus *Maesa,* is three times more effective than diclofenac sodium as an anti-inflammatory and anti-arthritic drug [51].

##### Histopathological Findings

**Figure 4:**
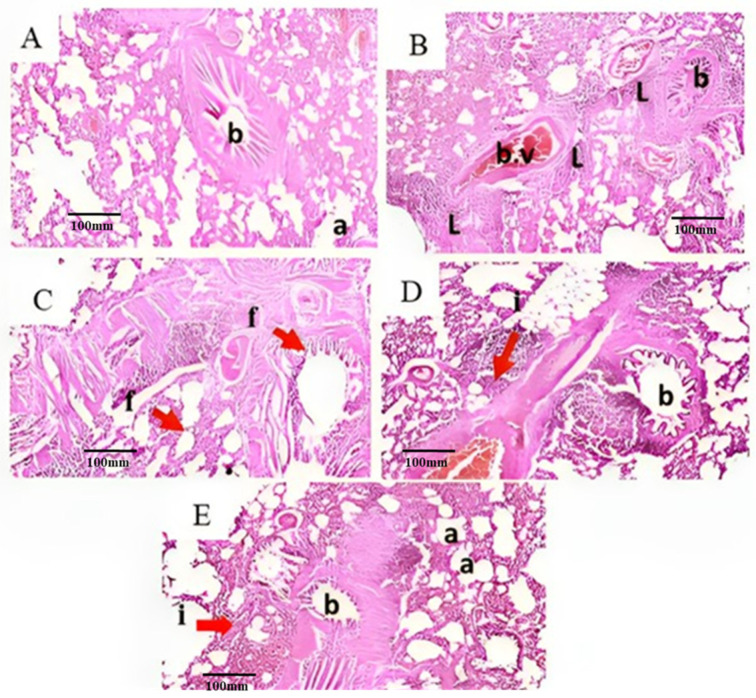
Effect of treatment with ME on PD-induced lung histopathological anomalies. (**A**) Lung sections from normal control rats showed average bronchioles with normal histological features, (**B**,**C**) Lung sections from the PD-treated group showing severe focal lymphoid aggregation, blood vessel dilatation and congestion as well as fibrotic changes (arrows). (**D**) Lung sections from rats treated with PD and ME 25 mg/kg, showing moderate infiltration of inflammatory cells (arrows). (**E**) Lung sections from rats treated with PD and ME 50 mg/kg, showing mild inflammatory infiltration (arrows), indicating dose-dependent protection against PD-induced lung injury.

No histopathological alterations were detected, and the normal histological structure of the bronchiole (b) with the surrounding air alveoli (a) were recorded in group of control rats (Figure 4A). The peribronchiolar tissue showed focal lymphoid cells aggregation (L) with dilatation of the blood vessels (b.v) associated with fibroblastic cells (f), and aggregation surrounding the bronchioles as well as in between the obliterated air alveoli in the group of rats experimentally-inducted with PD (Figure 4B,C). The peribronchiolar tissue showed moderate inflammatory cell (i) aggregation in the group of experimentally-inducted rats treated with ME (25 mg/kg) (Figure 4D). However, mild inflammatory cell aggregation was observed in the peribronchiolar tissue, and we found a few lymphoid cell aggregations in the group of experimentally-inducted rats treated with ME (50 mg/kg) Figure 4E. From these histopathological findings, we can conclude that the use of ME at a higher concentration (50 mg/kg) provides better lung protection than that produced by the lower concentration (25 mg/kg). ME reduces the histopathological changes caused by the exposure to PD which might be due to the high content of phenolics and flavonoids. Phenolic and flavonoid compounds have been reported to be able to markedly reduce lung histopathological changes [50].

All of these results confirmed the lung-protecting effect of the ethanolic extract of *Maesa indica.* This plant is rich in secondary metabolites, especially those which have antioxidant effects, such as phenolic and flavonoid compounds [5]. In this study, we identified 26 flavonoid compounds and 8 phenolic compounds. Most of these identified compounds have confirmed antioxidant capabilities and can prevent lung injury. Chlorogenic acid and phenolic acid were confirmed to have a potential protective effect against various toxicities like metals, pesticides, natural toxins, and pharmaceuticals. Mechanistic evaluations showed that inhibition of oxidative stress, free radical scavenging, apoptosis pathways, and decreasing the inflammatory responses are among the beneficial mechanisms mediated by chlorogenic acid [53]. Another study concluded that chlorogenic acid elevates the activity of SOD and the level of GSH and decreases the production of ROS and the accumulation of MDA [54]. Quercetin has been said to suppress lung inflammation in mice and to have an antioxidant effect, because the intratracheal administration of quercetin decreases the wet lung-to-body weight ratio (an index of pulmonary edema that is correlated with the severity of a lung injury) [55]. Apigenin administration reduces the biochemical parameters of oxidative stress and inflammation, improves oxygenation, and decreases lung edema in acute lung injuries in mice by inhibiting inflammation and oxidative stress [56]. According to [57], apigenin, quercetin, and luteolin were confirmed to have antioxidant effects. Kaempferol has proved to be a strong antioxidant, and it could significantly reduce the damage caused by lung ischemia-reperfusion injuries by inhibiting the release of inflammatory factors including interleukin lung ischemia-reperfusion injury and tumor necrosis factor α into the broncho-alveolar lavage fluid and reducing oxidative stress reactions [58]. In a lipopolysaccharide (LPS)-induced acute lung injury, rutin pretreatment prevented polymorphonuclear granulocyte infiltration into broncho-alveolar lavage fluid in addition to histological alterations in lung tissues. Furthermore, rutin reduced LPS-induced inflammatory responses in a concentration-dependent manner, including enhanced proinflammatory cytokine release and lipid peroxidation. Moreover, rutin reduces the effects of LPS on the activity of antioxidative enzymes such as catalase, glutathione peroxidase, hemeoxygenase-1, and superoxide dismutase [59]. The lung-protection effect of catechin and quercetin was detected during an assay of their effects on chlorpyrifos-induced lung toxicity in male rats. As a result, glutathione-S-transferase and glutathione peroxidase activities significantly increased, while MDA and SOD activities significantly decreased. Light microscopic studies revealed that histopathological changes were milder in animals treated with catechin or quercetin than in a control group [60]. Ref. [61] Explored the effect of naringenin on lipopolysaccharide (LPS)-induced acute lung injury in mice and the supposed mechanism. The results proved that naringenin pretreatment increased the rate of survival, enhanced histopathologic changes, attenuated pulmonary edemas and lung vascular leaks, and downregulated ROS level. In addition, naringenin pretreatment reduced the total and phosphorylated protein levels of phosphatidylinositol 3-hydroxy kinase (PI3K) and AKt. Pretreatment with rosmarinic acid strongly decreases lung injury caused by diesel exhaust particles (DEP) through its antioxidative and anti-inflammatory activities. Oral treatment of mice with rosmarinic acid inhibited DEP-caused lung injuries and edemas [62]. Ref. [63] Investigated the protective effect of caffeic acid phenethyl ester on acetylsalicylic-acid-induced lung injury in rats. The study concluded that caffeic acid phenethyl ester administration ameliorated the inflammation and eosinophil accumulation in the pulmonary interstitium in a histopathological manner, and it provided its lung-protecting effect by reducing oxidative damage and inflammation. Cinnamic acid was confirmed as a lung-protective compound effective against histopathological damage induced by methotrexate in rats. This was associated with parallel improvements in measured oxidative, inflammatory, and fibrotic parameters [64].

As mentioned before, alkaloids are strong therapeutic compounds, and they have many reported biological properties, including antimitotic, anticancer, anti-inflammatory, analgesic, antibacterial, antifungal, local analgesic, pain-relieving, antioxidant, antiparasitic, anti-plasmodic, antibacterial, anti-HIV, and as a local anesthetic [39]. The total alkaloids of the *Aconitum tanguticum* plant were found to have protective effects on acute lung injury induced by lipopolysaccharide in rats. The alkaloids significantly reduced the lung wet/dry ratio, reduced lung histopathological changes, decreased the nuclear factor kappa B (NF-κB) activation, downregulated inflammatory cell infiltration, and reduced vascular leakage and inflammatory cytokine release [65]. In lipopolysaccharides-induced acute lung injury in rats, total alkaloids decreased the levels of neutrophils, the number of WBCs, the levels of albumin, alkaline phosphate, and lactate dehydrogenase in broncho-alveolar lavage fluid (BALF), and increased the content of albumin in serum. It also improved SOD activity in lung, serum, and BALF, increased nitric oxide levels, and decreased MDA levels in lungs. Total alkaloids also inhibited the production of the inflammatory cytokines TNF-α and IL-8 in BALF and in the lung tissues. Histopathological examination showed that alkaloids suppressed histopathological changes caused by acute lung injury [66]. The two identified alkaloids, caffeine and trigonelline, were proven to have antioxidant properties, increase SOD activity and GSH levels, and decrease ROS production and MDA accumulation [67].

A variety of natural compounds can target cell-signaling pathways that promote beneficial activity against respiratory diseases. Natural products, including flavonoids, phenolics, alkaloids, and terpenes are at the same time a treasure trove of essential chemotherapeutics that produce desirable effects against respiratory diseases and lung injury. They also facilitate the development of novel drug systems by providing pharmacophores suitable for optimal effects on target pathways associated with the development of respiratory diseases [68]. This concept suggests the synergistic effect of ME phenolics, flavonoids, and alkaloid effects in lung protection, side by side with the approved lung protection of many of the compounds in ME identified in this study. 

## 3. Materials and Methods

### 3.1. Plant Materials

*M. indica* Roxb. Sweet aerial parts before blooming were collected from the EL-MAZHAR botanical garden, El-Barageel, Giza, Egypt. The plant was verified by Therese Labib, a senior botanist at El-Orman Botanical Gardens and a plant taxonomy consultant at the Ministry of Agriculture, Egypt. The specimen voucher with the number 19.06.2022 was kept in the Pharmacognosy Department herbarium of the Faculty of Pharmacy, Cairo University. The plant material was allowed to air dry before being pounded into a coarse powder, put in an appropriate amber glass, sealed in an airtight container, and kept at room temperature.

### 3.2. Extraction of Plant Materials

Five hundred grams of the powdered MI were extracted using one liter of 70% ethanol (three times until complete extraction) at room temperature. The ethanol was evaporated using rotavapor until dryness giving about 6 gm of thick extract (ME). 

### 3.3. Metabolic Profiling Using T-TOF LC/MS/MS

#### 3.3.1. Chemicals

Methanol and formic acid (LC grade) were supplied from Fisher Scientific (Hampton, UK). Acetonitrile and ammonium formate (LC grade) were purchased from Sigma-Aldrich (Darmstadt, Germany). Millipore water was used (Burlington, MA, USA).

#### 3.3.2. Instrument

LC-MS/MS was performed using an ExionLC™ AC system coupled with an AB Sciex TripleTOF 5600+ mass spectrometer (SCIEX, Toronto, ON, Canada). Non-targeted screening was applied using electron spray ionization (ESI) as the ionization probe. Data processing and peak identification were performed using an MS-DIAL 4.6 with the Respect library database.

#### 3.3.3. Chromatographic Conditions

The separation was performed using an X select HSS T3 Column (2.1 × 150 mm, 2.5 µm). The mobile phases consisted of two eluents A: 5 mM ammonium formate buffer pH 8 containing 1% methanol; B: acetonitrile. The mobile phase elution was programmed as follows: 10% B from 0–1 min, 10–90% B from 1–21 min, 90% B from 21–25 min, 90–10% B from 25–25.01 min, and finally holds for three minutes at 10% B. The flow rate was 0.3 mL/min and the injection volume was 10 µL. Negative ionization mode was applied with a workflow EMS-IDAEPI and a scan from 50 to 1000 Da. For MS1, the following parameters were adjusted: curtain gas: 25 psi; IonSpray voltage: −4500, positive mode IonSpray voltage was 4500; source temperature: 500 °C; ion source gas 1 & 2 were 45 psi while in the case of MS2, a scan from 50 to 1000 Da was also adjusted with a declustering potential: −80; collision energy: −35; collision energy spread: 15.

#### 3.3.4. Sample Preparation

Prepare a reconstitution solvent composed of Water: Methanol: Acetonitrile (50:25:25) *v*/*v*. 50 mg of the sample is dissolved in 1 mL of the reconstitution solvent. Vortex for 2 min followed by ultra-sonication for 10 min. Centrifuge for 10 min at 10,000 rpm. Dilute 50 µL of stock solution to 1000 µL by reconstitution solvent. The injected concentration is 2.5 µg/µL. Inject 10 µL on negative mode. Inject 10 µL reconstitution solvent as a blank sample.

### 3.4. The In-Vivo Study

#### 3.4.1. Acute Toxicity Study

For this investigation, male and female Swiss mice weighing 20–30 gm were procured from the animal house laboratory at the National Research Centre (NRC), Cairo, Egypt. Mice were kept in a hygienic laboratory environment for seven days before the start of the biological experiment (adaptation period), held in a well-ventilated box at 22 ± 2 °C for a 12-h lighting and darkness cycle. A natural baseline diet was given to the mice. Diets and water were provided freely. They were managed according to the animal testing guidelines approved by the Ethical Committee of Medical Research, NRC, Cairo, Egypt. Acute toxicity was carried out in compliance with the guidelines of the World Health Organization (WHO) for assessing the safety and efficacy of herbal remedies. Forty Swiss mice were divided into four groups (ten for each group). Groups one and two: control male and female mice were orally administered with saline. Groups three and four: male and female mice were orally administered with a single dose of ME in graded doses up to 5 g/kg. The animals were observed daily for signs of behavioral changes for two weeks [69].

#### 3.4.2. Investigation of Pulmonary Protection Activity

##### Animals

Adult male Wister albino rats (150–200 g) were provided by the Animal House of the National Research Centre (Cairo, Egypt). The rats were group-housed under temperature- and light-controlled conditions (22 ± 2 °C under a 12 h light/dark cycle) and had free access to standard laboratory rodent chow and water. The animal experiments were performed according to NRC and recommendations in the Guide for the Care and Use of Laboratory Animals of the National Institutes of Health (NIH No. 85:23 revised 1985). This study was approved by the Ethics Committee of the Faculty of Pharmacy, Cairo University, Egypt [Serial number of the protocol: MP (2992)].

##### Drugs, Chemicals and Kits

PD was purchased from (Santa Cruz, CA, USA). MDA and GSH were determined using a Biodiagnostic kit, Giza, Egypt. AKt and PI3K were determined using ELISA kits procured from (Sunlong Biotech Co., Ltd., Hangzhou, China).

##### Experimental Design of Pulmonary Damage

Male Wister albino rats were randomly allocated into four groups (*n* = 8) as follows: Control group: Rats were injected with a single intranasal (i.n) instillation of normal saline and received normal saline orally for ten consecutive days. PD group: Rats were injected with a single i.n instillation of PD (2 mg/kg in a volume of 500 μL) [10]. ME groups: rats were administered with ME (25 & 50 mg/kg) once daily for seven consecutive days before the intranasal (i.n) instillation of PD.

#### Biochemical Analysis

At the end of the experimental period, the animals were anesthetized with pentobarbital sodium and sacrificed by decapitation. One lung from each rat was immediately dissected out, washed with ice-cooled physiological saline, and homogenized in phosphate-buffered saline (PBS) (pH 7.4) as 20% (*w*/*v*) for the biochemical measurements [70]. The other lung was kept for histopathological assessment.

##### Estimation of MDA and GSH

Serum was used for the estimation of reduced glutathione (GSH) and malondialdehyde (MDA) levels. The GSH estimation method is based on the reduction of 5,5 dithiobis (2-nitrobenzoic acid) (DTNB) with reduced GSH to produce a yellow compound. The reduced chromogen is directly proportional to GSH concentration, and its absorbance can be measured at 405 nm [71]. MDA measuring depends on the formation of MDA as an end product of lipid peroxidation. The MDA reacts with thiobarbituric acid, producing a thiobarbituric acid reactive substance (TBARS), a pink chromogen, which can be measured spectrophotometrically at 532 nm. An MDA standard was used to construct a standard curve against which readings of the samples were plotted [10]. 

##### Estimation of AKt and PI3K

The contents of AKt and PI3K in each lung were determined using ELISA kits. Standards and samples (50 μL) were pipetted into wells with immobilized antibodies specific for rat AKt and PI3K and were then incubated for 30 min at 37 °C. After incubation and washing, horseradish peroxidase-conjugated streptavidin was pipetted into the wells and incubated for 30 min at 37 °C, which were washed once again. Tetramethylbenzidine (TMB) substrate solution was added to the wells and incubated for 15 min at 37 °C; a color developed proportionally to the amount of AKt and PI3K bound. Color development was discontinued (stop solution) and after 10 min the color intensity was measured at 450 nm [72].

Then the concentration of each parameter (pg/g) and the % change in concentration between groups are calculated as follows:

% change = 100 − (conc of treatment/mean conc of diseased) × 100

##### Histological Examination

The dissected lungs of different groups were fixed in 10% formalin. Fixation for one or two days was followed by dehydration in ascending grades of alcohol (70%, 90%, and three changes in absolute alcohol), clearance with xylene, impregnation in three successive changes in soft paraffin at 50 °C, and finally embedded in paraffin wax to obtain solid blocks containing the tissue. Serial transverse sections of 7 μm thick were cut. Paraffin sections were mounted on glass slides covered by albumin glycerin and then stained with Haematoxylin and Eosin. Hematoxylin and Eosin sections were evaluated qualitatively under light microscopy.

#### 3.4.3. Statistical Analysis

All the values are presented as means ± SD. Data of this study were evaluated by one-way analysis of variance followed by Tukey’s multiple comparisons test. Graphpad Prism software, version 5 (Iglesia Ni Cristo., San Diego, CA, USA) was used to carry out these statistical tests. The difference was considered significant when *p* < 0.05.

## 4. Conclusions

The present study investigated the chemical profile of 70% *Maesa indica* ethanolic extract and identified 56 compounds via UPLC-ESI-TOF-MS in both negative and positive ESI ionization modes. The identified compounds include 26 flavonoids, 17 amino acids, 8 phenolics, 3 sugars, and 2 alkaloids. The study also investigated the lung-protective capacity of the plant ethanolic extract against potassium dichromate-induced pulmonary damage in rats. The results confirmed the lung-protection capacity of ME. The ME inhibited the histopathological changes caused by potassium dichromate. Furthermore, the administration of ME caused a significant increase in GSH, AKt, and PI3K levels and significant decrease in MDA level. Considering these results, it can be inferred that *Maesa indica* is a potent lung-protective plant and can alleviate potassium dichromate-induced pulmonary damage. Further investigation and clinical trials are needed to assess the exact mechanism of action of ME and to determine if these results can be applied to human diseases.

## Figures and Tables

**Table 1:** Metabolites tentatively identified in the 70% ethanol extract of (*Maesa indica*) via UPLC ESI-TOF-MS in both negative/positive ESI ionization modes.

No.	RT (min)	Mol. Ion *m*/*z*		Identified Compound	Molecular Formula	Error (ppm)	Fragment Ions
		[M − H]−	[M + H]+				
Flavonols							
3.	1.22153	319.0688	317.0546	Myricetin	C_15_H_10_O_8_	1.8	317.0546, 281.1009, 225.0102
7.	1.33837	477.0106		Quercetin-3-Glucuronide	C_21_H_18_O_13_	−1.3	477.0106, 301.0204, 271.0723, 242.9609, 163.0403, 151.0251
10.	1.41537	433.0459		Quercetin-3-D-xyloside	C_20_H_18_O_11_	−0.8	433.0459, 301.0204
29.	4.7067	593.1576		Kaempferol-7-neohesperidoside	C_27_H_30_O_15_	−1.8	593.1576, 285.0364
30.	4.90885	609.1462	611.1603	Rutin	C_27_H_30_O_16_	−1	611.1603, 303.110, 143.03
34.	5.39382	315.1088		3′-methoxy-4′,5,7-trihydroxyflavonol	C_16_H_12_O_7_	1	315.1088, 300.236
36.	6.16062	463.0924		Quercetin-4′-glucoside	C_21_H_20_O_12_	−5.9	463.0924, 301.0264
37.	6.22428	447.0976		Quercitrin (Quercetin-3-*O*-rhamnoside)	C_21_H_20_O_11_	−7.9	477.0996, 429.2153, 401.1227, 301.2084
41.	6.62018	447.0948		Kaempferol-3-*O*-glucoside	C_21_H_20_O_11_	−1.7	447.0948, 285.0409, 255.025
42.	6.73458	477.0996		Isorhamnetin-3-*O*-glucoside	C_22_H_22_O_12_	7.2	477.0996, 315.0204, 301.110
43.	7.07877	301.0503	303.046	Quercetin	C_15_H_10_O_7_	7.3	303.046, 285.1349, 153.2648, 137.0592
44.	7.09293		465.1005	Hyperoside (Quercetin 3-galactoside)	C_21_H_20_O_12_	2.3	465.1005, 303.0500
46.	7.7529		301.0998	3 5 7-trihydroxy-4′-methoxyflavone (Diosmetin)	C_16_H_12_O_6_	4.3	301.0998, 283.0971, 255.0724
Flavones							
12.	1.44153	417.0578		Kaempferol-3-*O*-alpha-L-arabinoside	C_20_H_18_O_10_	−0.8	417.0578, 285.0406, 284.0327
35.	5.74847	445.0771		Baicalein-7-*O*-glucuronide	C_21_H_18_O_11_	0.4	445.0771, 269.045, 175.0244
38.	6.23795	591.1369		Acacetin-7-*O*-rutinoside (Linarin)	C_28_H_32_O_14_	−0.8	591.1369, 445.0101, 283.0318
39.	6.3527	285.0396		Luteolin	C_15_H_10_O_6_	2.5	285.0396, 163.6529, 151.005
40.	6.61593	415.1646		Puerarin	C_21_H_20_O_9_	−6.7	415.1646, 295.0405, 253.1208
47.	7.8312	431.0983		apigenin-7-*O*-glucoside	C_21_H_20_O_10_	0.8	431.0983, 269.0423, 268.0355
51.	8.86035		269.0807	Formononetin	C_16_H_12_O_4_	1.8	269.0807, 237.0812, 213.0736
52.	10.0624	269.0419		Apigenin	C_15_H_10_O_5_	3.2	269.0419, 117.0327
54.	12.3865		433.114	Apigenin 8-C-glucoside (vitexin)	C_21_H_20_O_10_	−0.8	433.114, 415.1061, 313.0744
Flavanones							
1.	1.182367	271.0123		Naringenin	C_15_H_12_O_5_	0.1	271.0123, 151.005, 119.051, 107.014
45.	7.487216	301.1187		Hesperetin	C_16_H_14_O_6_	1.9	301.1187, 283.1092, 161.0413
53.	11.38955		289.1188	3′ 4′ 5 7-tetrahydroxyflavanone (fustin)	C_15_H_12_O_6_	2.1	271.0123, 151.005, 119.051, 107.014
Flavanols							
34.	5.53515	289.011		catechin	C_15_H_14_O_5_	2.1	289.011, 245.098
Phenolic acids							
19.	1.49335	353.0888		Chlorogenic acid	C_16_H_18_O_9_	0.8	353.0888, 191.0546, 173.0496, 135.100
24.	2.353867		149.0598	*Trans*-Cinnamic acid	C_9_H_8_O_2_	−0.3	149.0598, 131.0492, 105.0540
26.	3.325267	137.0248		P-hydroxybenzoic acid	C_7_H_6_O_3_	−0.9	137.0239, 93.0341
31.	4.95135	153.0183		3,4-Dihydroxybenzoic acid (Protocatechuic acid)	C_7_H_6_O_4_	3.7	153.0183, 109.0297, 108.0218
48.	7.857533	359.0174		Rosmarinic acid	C_18_H_16_O_8_	0.4	359.0174, 315.0241, 161.0540, 135.0709
49.	8.054216		411.1733	γ-Tocotrienol (vitamin E)	C_28_H_42_O_2_	1.2	411.1733, 409.8878, 242.12191, 100.4870
50.	8.084617		179.1066	Daphnetin	C_9_H_6_O_4_	0.1	179.1066, 135.03, 77.0404
55.	14.40788	179.0549		Caffeic acid	C_9_H_8_O_4_	4.5	179.0549, 163.0349, 138.0005, 135.0644, 109.0569
Amino acids							
2.	1.205733		162.0765	Carnitine	C_7_H_15_NO_3_	0.5	162.0765, 85.0287, 72.421
5.	1.286033	146.0468		L-Glutamic acid	C_5_H_9_NO_4_	−0.9	146.0468, 128.0347, 102.0588, 100.0358, 91.0552
6.	1.293733		104.1054	N,N-Dimethylglycine	C_4_H_9_NO_2_	0.7	104.1054, 60.0829, 59.0735, 58.0663
8.	1.344233		156.0427	Histidine	C_6_H_9_N_3_O_2_	−0.9	156.0427, 110.0036, 93.0434, 83.0586, 68.9831
13.	1.4579		132.0641	*trans*-4-Hydroxy-L-proline	C_5_H_9_NO_3_	3.3	132.0641, 86.0656, 68.0452, 57.0568
14.	1.4670		133.0591	L-Asparagine	C_4_H_8_N_2_O_3_	2.9	133.0591, 116.0347, 87.0537, 74.0284, 70.0329
15.	1.47007		116.0712	L-Proline	C_5_H_9_NO_2_	1.3	116.0712, 70.0657
16.	1.4797		146.0919	L-β-Homoleucine	C_7_H_15_NO_2_	0.7	146.0919, 87.1103, 86.0969, 69.0715
17.	1.47985	116.0707		Norvaline	C_5_H_11_NO_2_	1.2	116.0707, 59.0301
20.	1.598517	130.0858		Hydroxy proline	C_5_H_9_NO_3_	4.8	130.0858, 113.04095
21.	1.660383		175.1184	L-Arginine	C_6_H_14_N_4_O_2_	−0.1	175.1184, 158.0913, 116.068, 70.0659
22.	1.903867		130.0483	L-5-Oxoproline	C_5_H_7_NO_3_	7.5	130.0483, 84.0805, 56.048
23.	2.049333	134.0461		Adenine	C_5_H_5_N_5_	2.5	134.0461, 107.0416
25.	2.374333		166.0866	L-Phenylalanine	C_9_H_11_NO_2_	−2	166.0866, 120.0792, 103.0544, 91.054, 79.0511
27.	3.403083	203.0845		L-tryptophan	C_11_H_12_N_2_O_2_	−0.3	188.0687, 170.0324, 159.0884, 142.0646, 132.0801
28.	4.472367	144.0445		L-β-Homoisoleucine	C7H15NO2	0.7	144.0445, 87.1103, 86.0969, 69.0715
56	15.9923		385.1516	S-Adenosyl-L-homocysteine	C_14_H_20_N_6_O_5_S	0.3	385.1516, 275.1985, 133.0981
Alkaloids							
11.	1.43257		138.052	Trigonelline	C_7_H_7_NO_2_	2.3	138.052, 110.0599, 94.0651, 92.0489
32.	5.00988		195.086	Caffeine	C_8_H_10_N_4_O_2_	3.2	195.086, 163.034, 138.005, 95.0816
Carbohydrates							
4.	1.26053	195.0521		Gluconate	C_6_H_12_O_7_	1.7	195.0521, 159.8957, 116.933, 99.9293, 76.9712
9.	1.35473	179.0557		D-tagatose	C_6_H_12_O_6_	0.6	179.0557, 89.0245, 71.0138, 59.0136, 43.0184
18.	1.48225	341.1109		Galactinol dihydrate	C_12_H_22_O_11_	−2.3	341.1073, 295.093, 179.061, 161.0456, 143.0326

## Data Availability

The data presented in this study are available on request from the corresponding author.

## Supplementary Materials

The following supporting information can be downloaded at: https://www.mdpi.com/article/10.3390/plants13030338/s1, Figure S1: MS/MS spectrum of peak 3: Myriceti; Figure S2: MS/MS spectrum of peak 7: Quercetin-3-Glucuronide; Figure S3: MS/MS spectrum of peak 10: Quercetin-3-D-xyloside; Figure S4: MS/MS spectrum of peak 39: kaempferol 7-neohesperidosid; Figure S5: MS/MS spectrum of peak 30: Rutin (Quercetin-*O*-rutinoside); Figure S6: MS/MS spectrum of peak 36: Quercetin-4′-glucoside; Figure S7: MS/MS spectrum of peak 41: Kaempferol-3-*O*-glucoside; Figure S8: MS/MS spectrum of peak 42: Isorhamnetin-3-*O*-glucoside; Figure S9: MS/MS spectrum of peak 43: Quercetin; Figure S10: MS/MS spectrum of peak 44: hyperoside (quercetin-3-*O*-galactoside); Figure S11: MS/MS spectrum of peak 46: 3, 5, 7-trihydroxy-4′-methoxyflavone (Diosmetin); Figure S12: MS/MS spectrum of peak 12: Kaempferol-3-O-alpha-L-arabinoside; Figure S13: MS/MS spectrum of peak 35: Baicalein-7-*O*-glucuronide; Figure S14: MS/MS spectrum of peak 38: Acacetin-*O*-rutinoside (linarin); Figure S15: MS/MS spectrum of peak 39: Luteolin; Figure S16: MS/MS spectrum of peak 47: apigenin-7-*O*-glucoside; Figure S17: MS/MS spectrum of peak 51: Formononetin; Figure S18: MS/MS spectrum of peak 39: Apigenin; Figure S19: MS/MS spectrum of peak 54: Apigenin 8-C-glucoside (vitexin); Figure S20: MS/MS spectrum of peak 1: Naringenin; Figure S21: MS/MS spectrum of peak 45: Hesperetin; Figure S22: MS/MS spectrum of peak 53: 3′ 4′ 5 7-tetrahydroxyflavanone; Figure S23: MS/MS spectrum of peak 34: Catechin; Figure S24: MS/MS spectrum of peak 19: Chlorogenic acid; Figure S25: MS/MS spectrum of peak 24: *Trans*-Cinnamate; Figure S26: MS/MS spectrum of peak 26: P-Hydroxybenzoic acid; Figure S27: MS/MS spectrum of peak 31: 3,4-Dihydroxybenzoic acid (Protocatechuic acid); Figure S28: MS/MS spectrum of peak 48: Rosmarinic acid; Figure S29: MS/MS spectrum of peak 55: Caffeic acid; Figure S30: MS/MS spectrum of peak 11: Trigonelline; Figure S31: MS/MS spectrum of peak 32: Caffeine.
