# Supplementary material for: Phytochemical Elucidation and Effect of Maesa indica (Roxb.) Sweet on Alleviation of Potassium Dichromate-Induced Pulmonary Damage in Rats
Source: Plants (Basel). 2024 Jan 23;13(3):338. doi: 10.3390/plants13030338 (PMC10857331; doi:10.3390/plants13030338)
Supplement: Supplementary file 1 [file plants-13-00338-s001.zip › plants-2788055-supplementary.pdf]

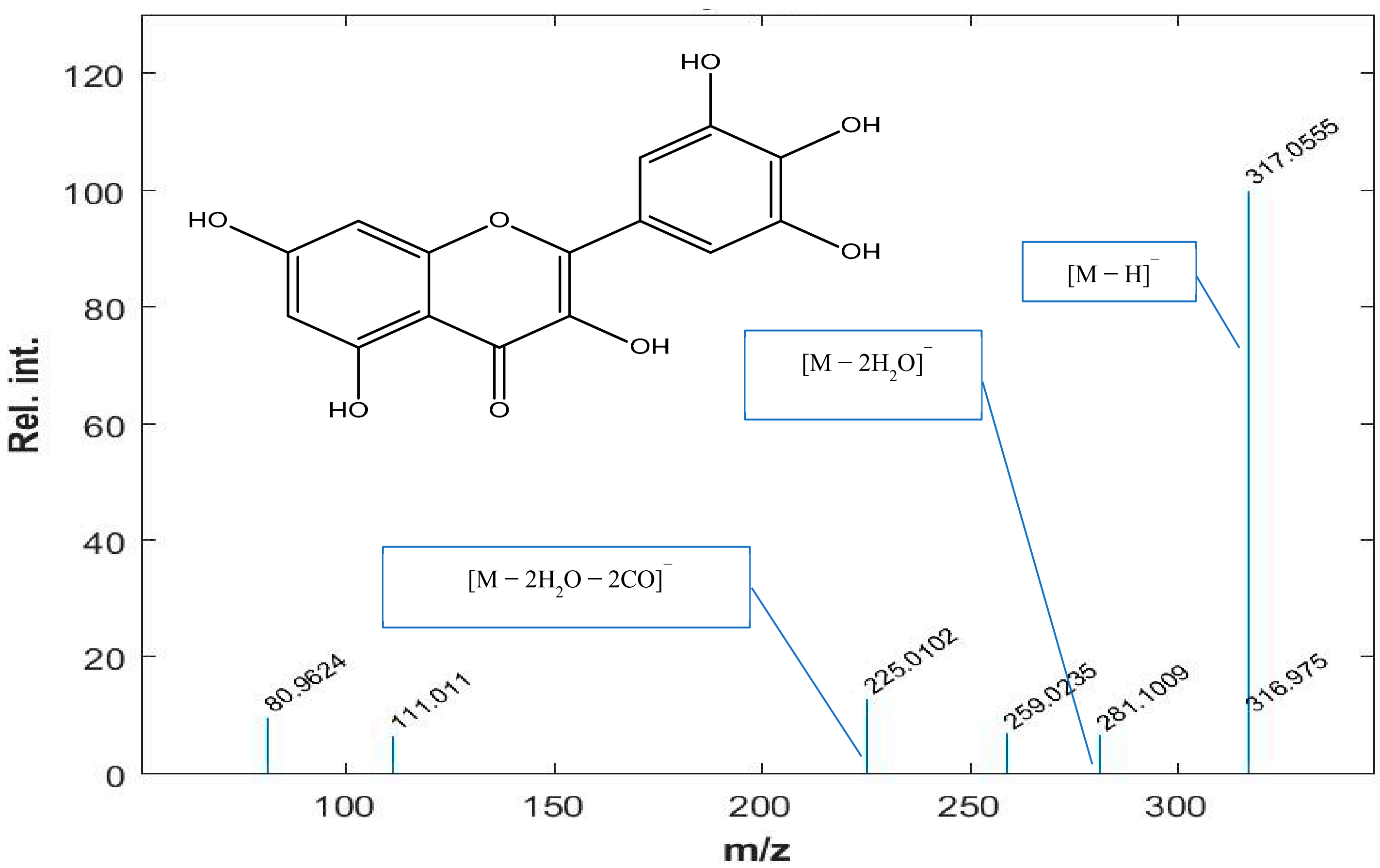

Figure S1. MS/MS spectrum of peak 3: Myricetin

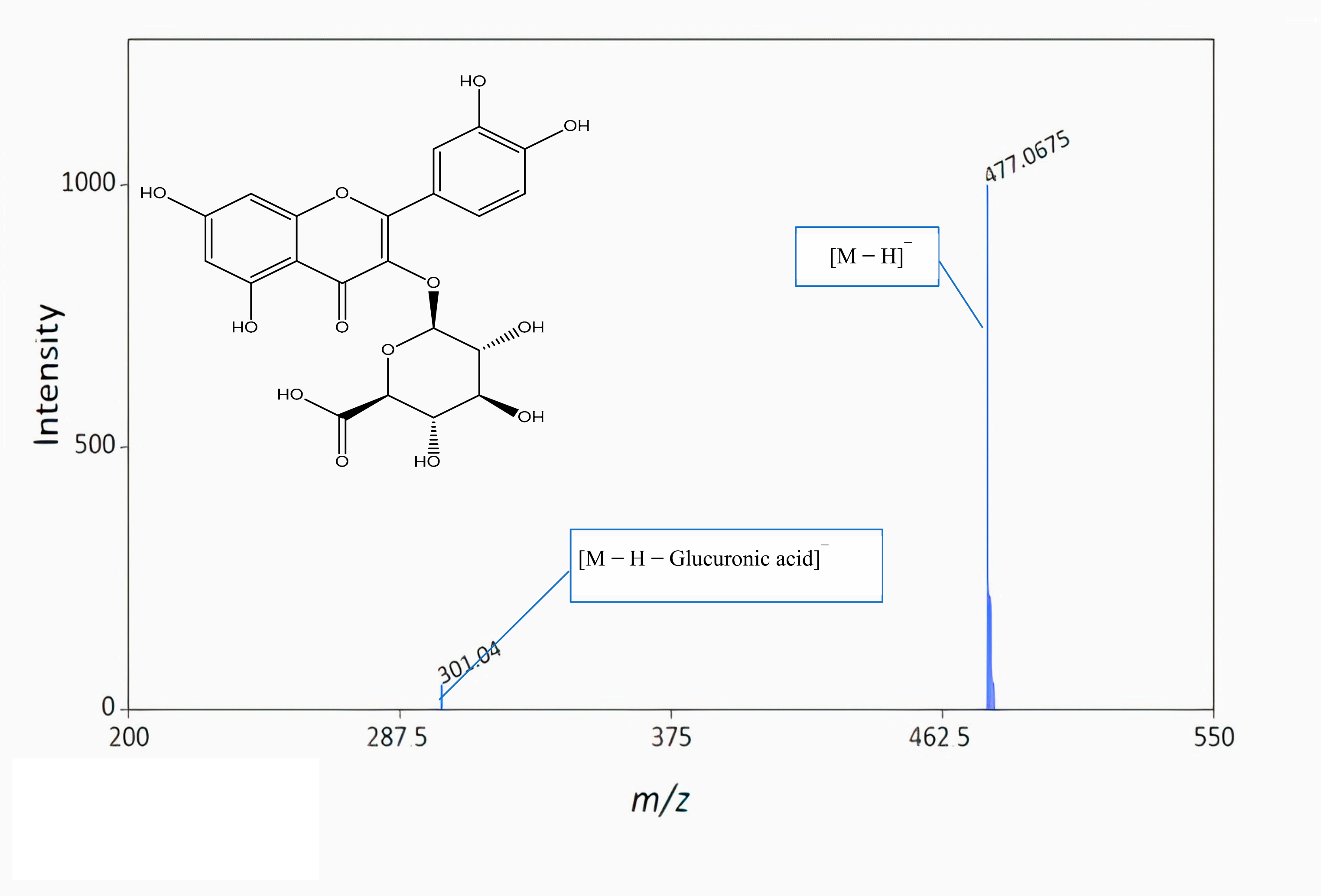

Figure S2. MS/MS spectrum of peak 7: Quercetin-3-Glucuronide

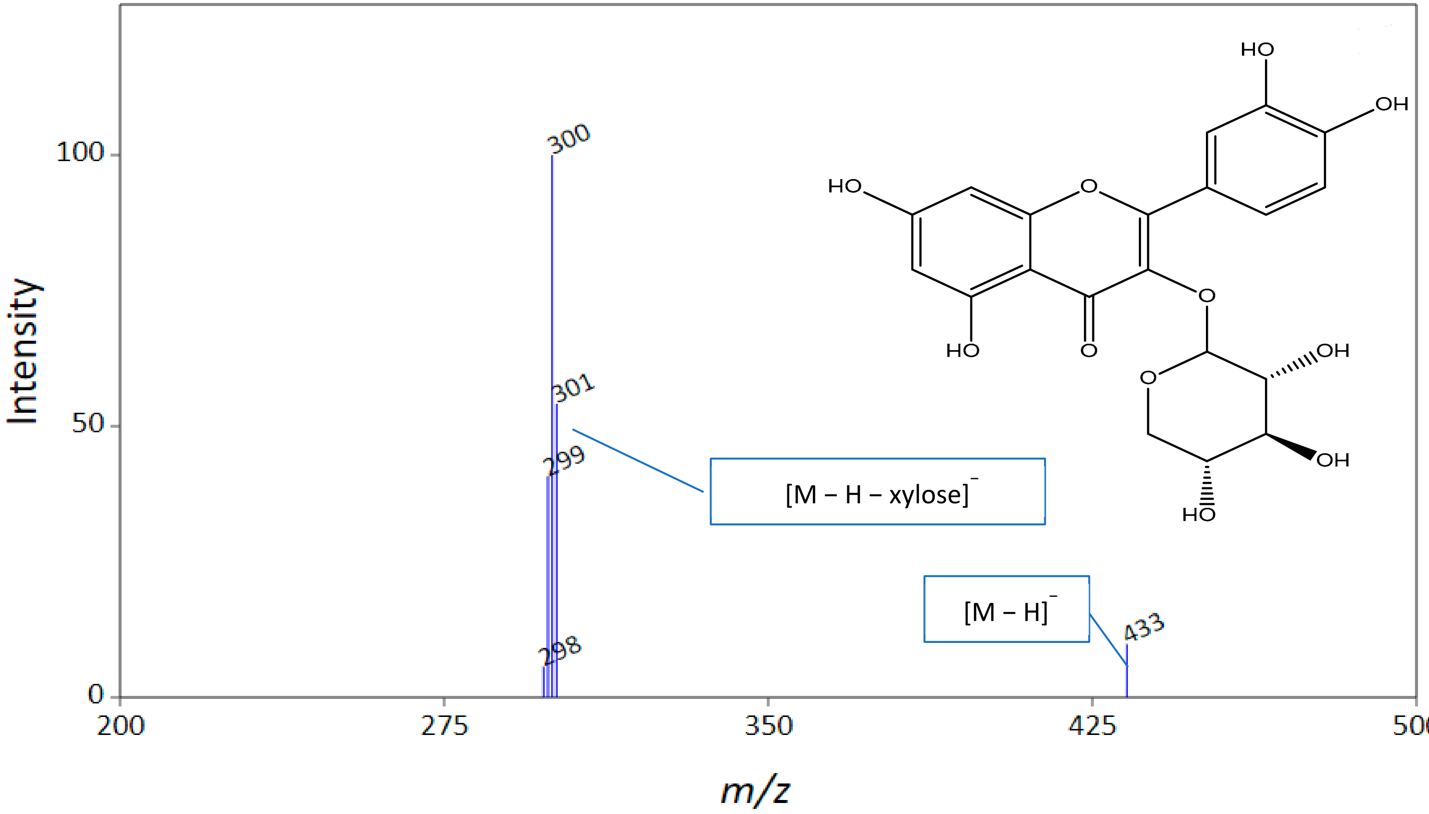

Figure S3. MS/MS spectrum of peak 10: Quercetin-3-D-xyloside

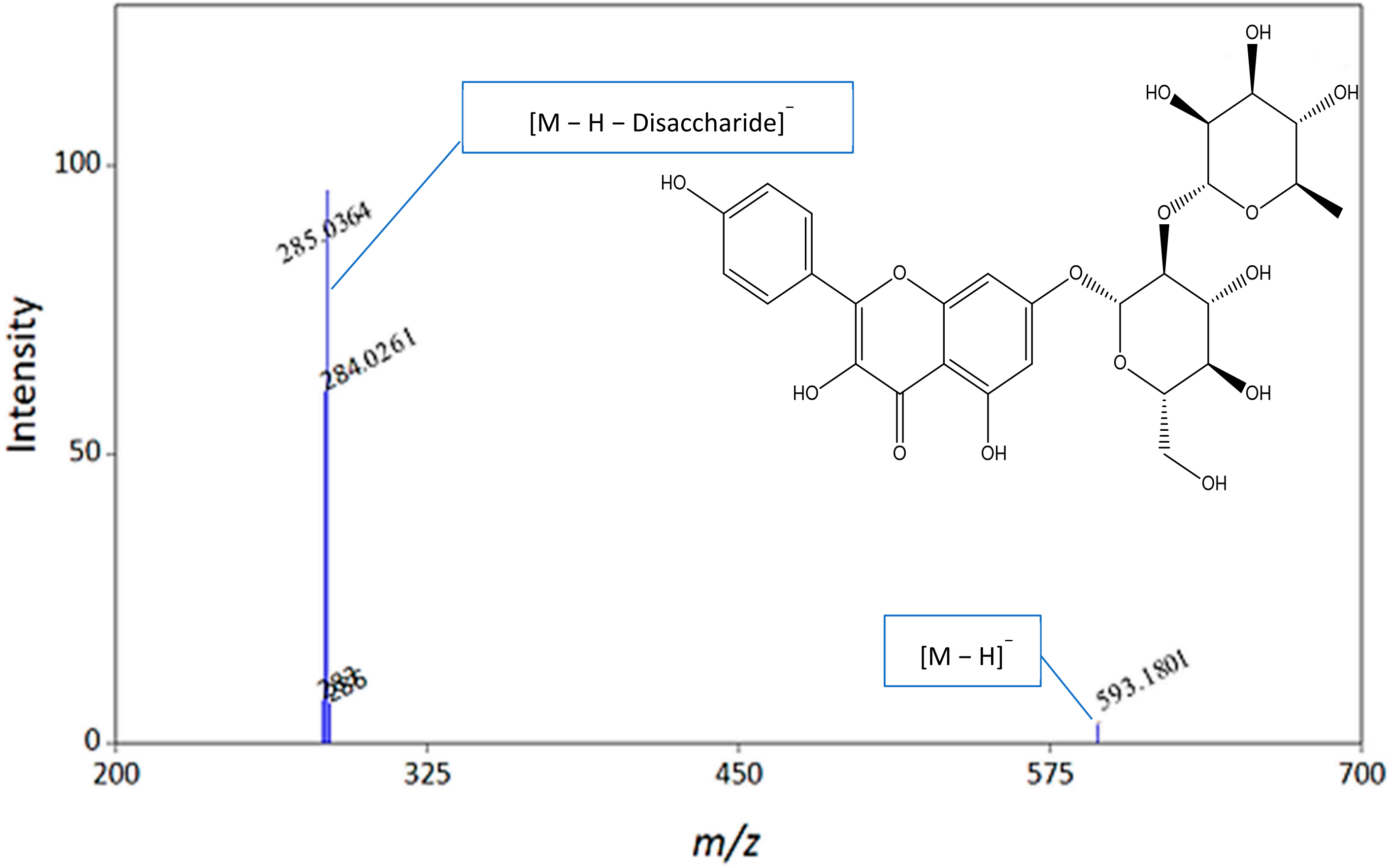

Figure S4. MS/MS spectrum of peak 39: kaempferol 7-neohesperidosid

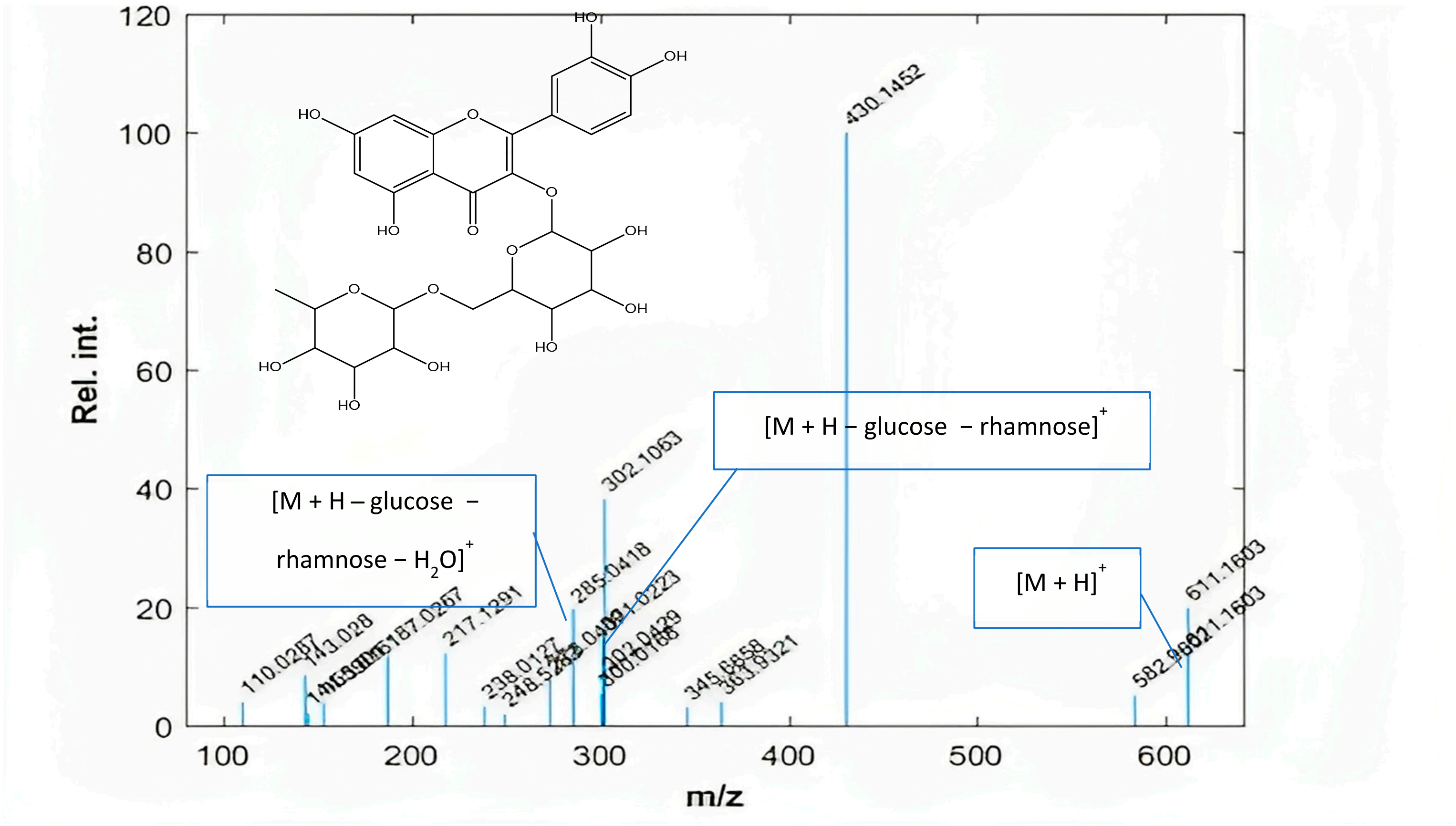

Figure S5. MS/MS spectrum of peak 30: Rutin (Quercetin-*O*-rutinoside)

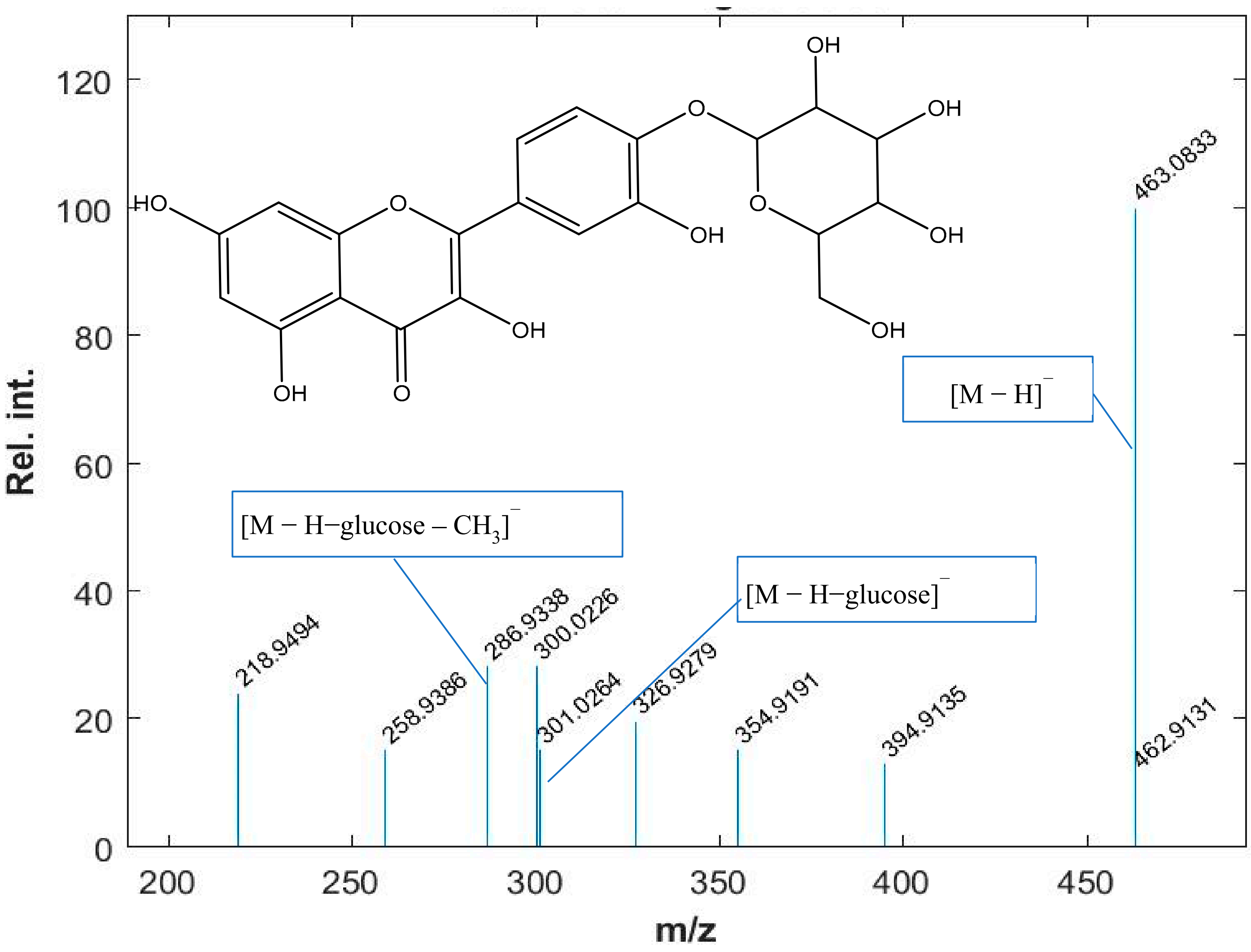

Figure S6. MS/MS spectrum of peak 36: Quercetin-4'-glucoside

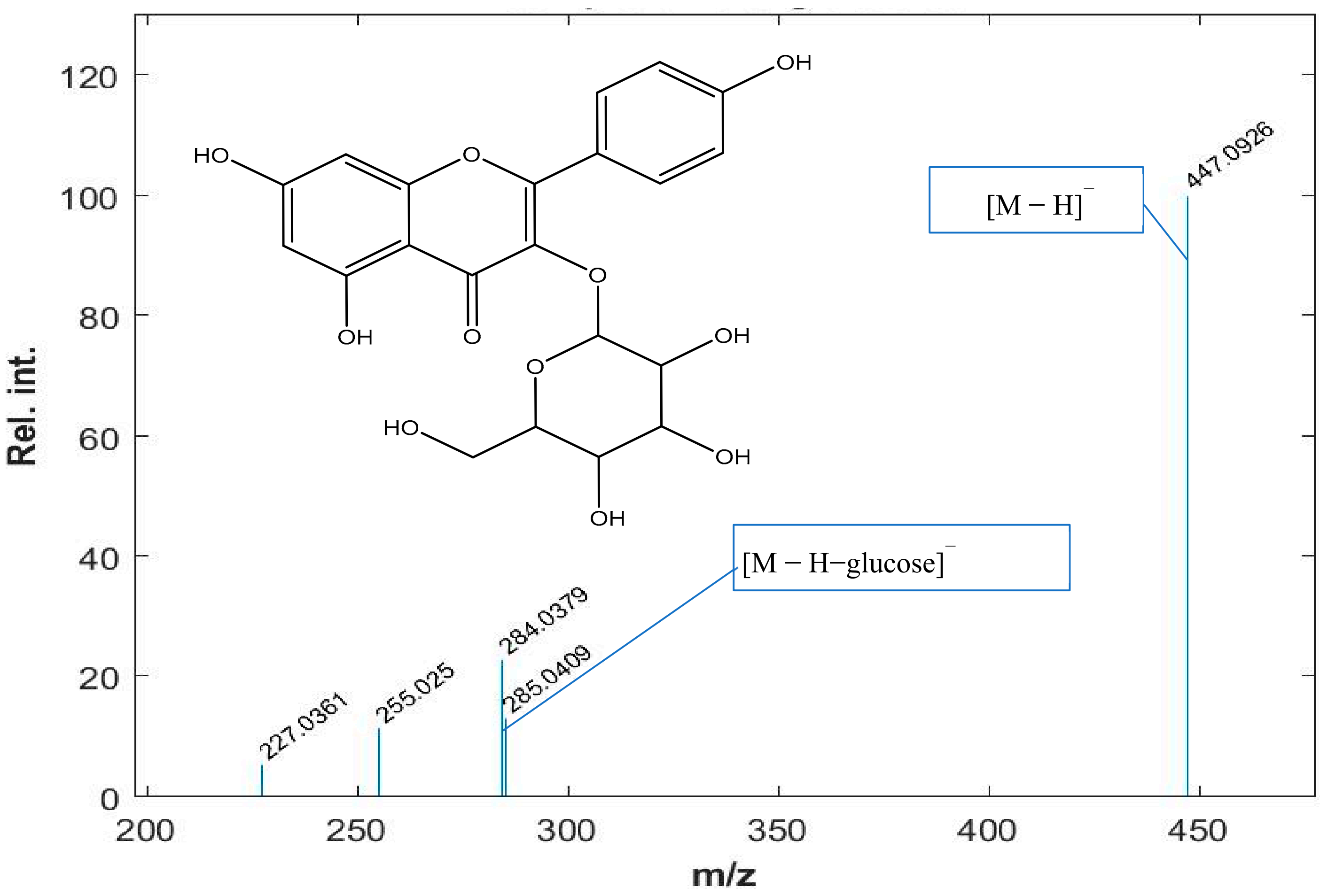

Figure S7. MS/MS spectrum of peak 41: Kaempferol-3-*O*-glucoside

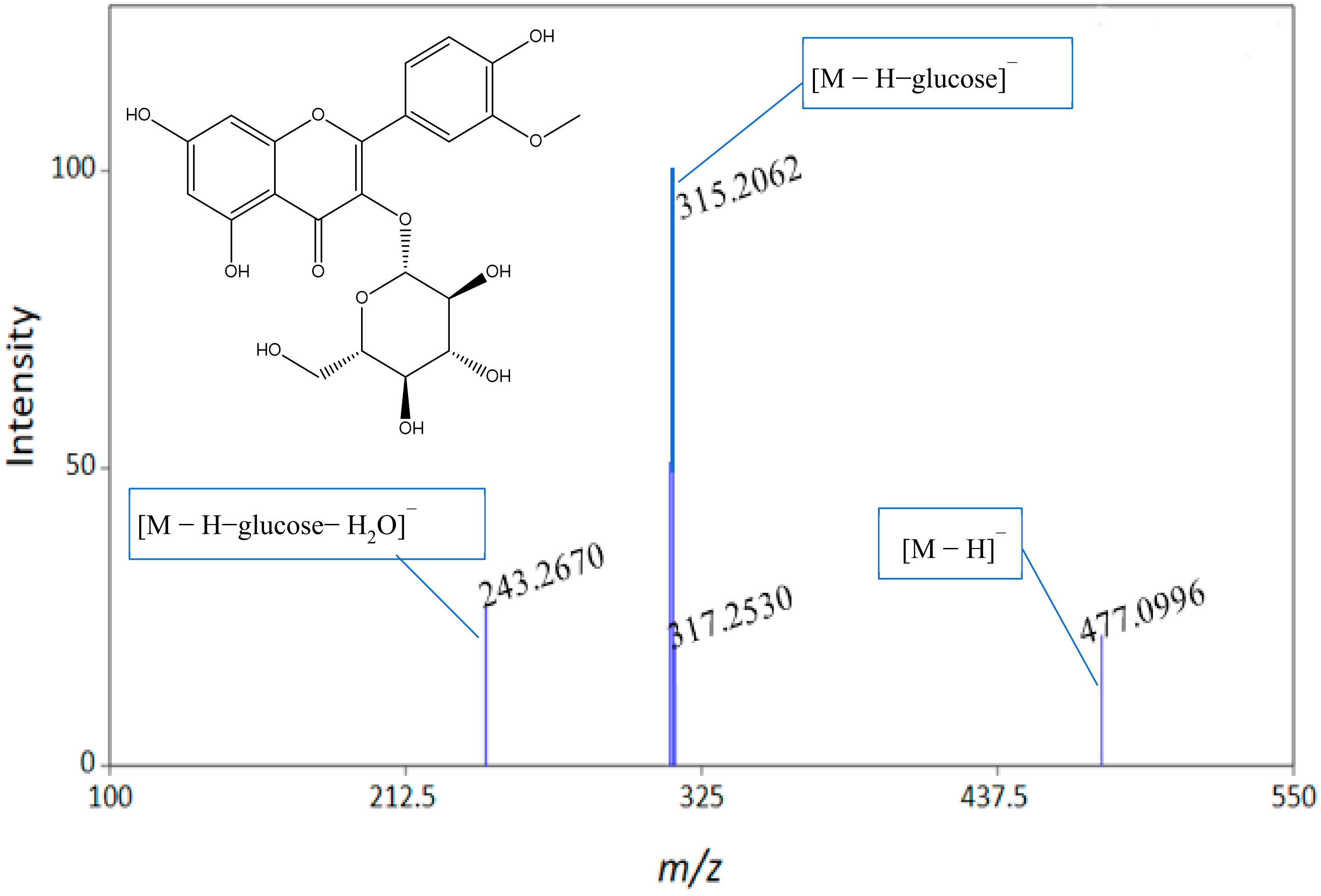

Figure S8. MS/MS spectrum of peak 42: Isorhamnetin-3-*O*-glucoside

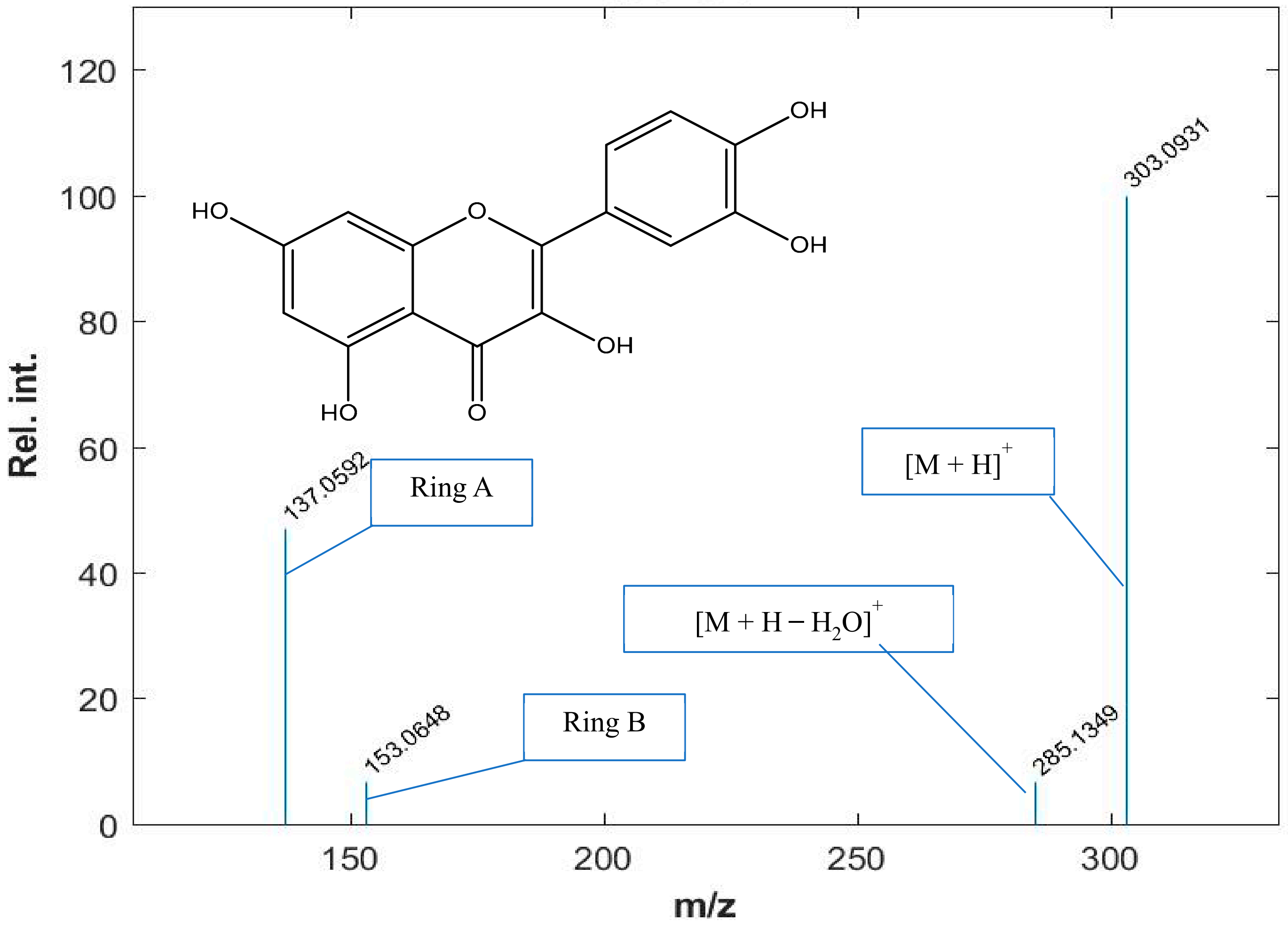

Figure S9. MS/MS spectrum of peak 43: Quercetin

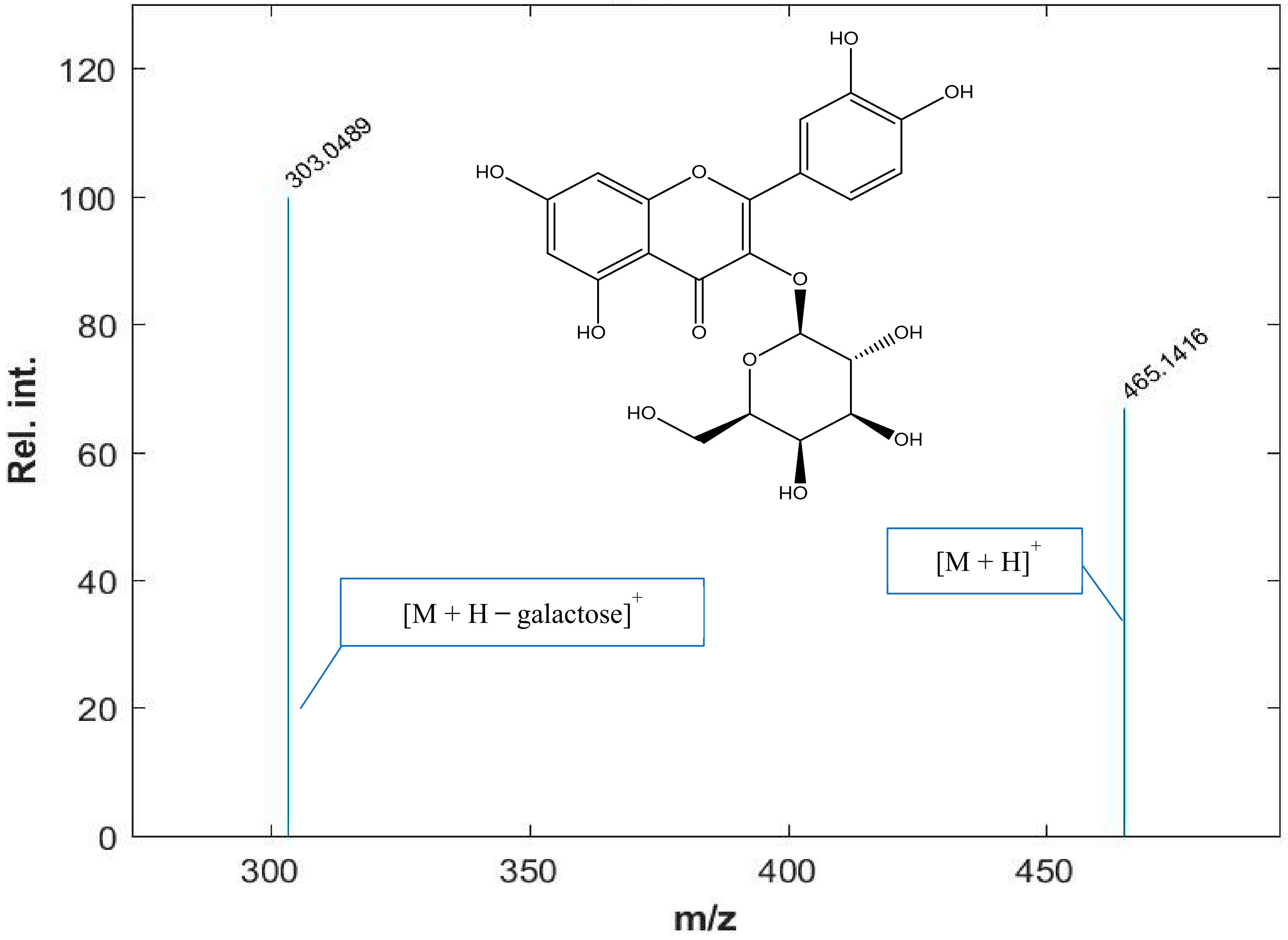

Figure S10. MS/MS spectrum of peak 44: hyperoside (quercetin-3-*O*-galactoside)

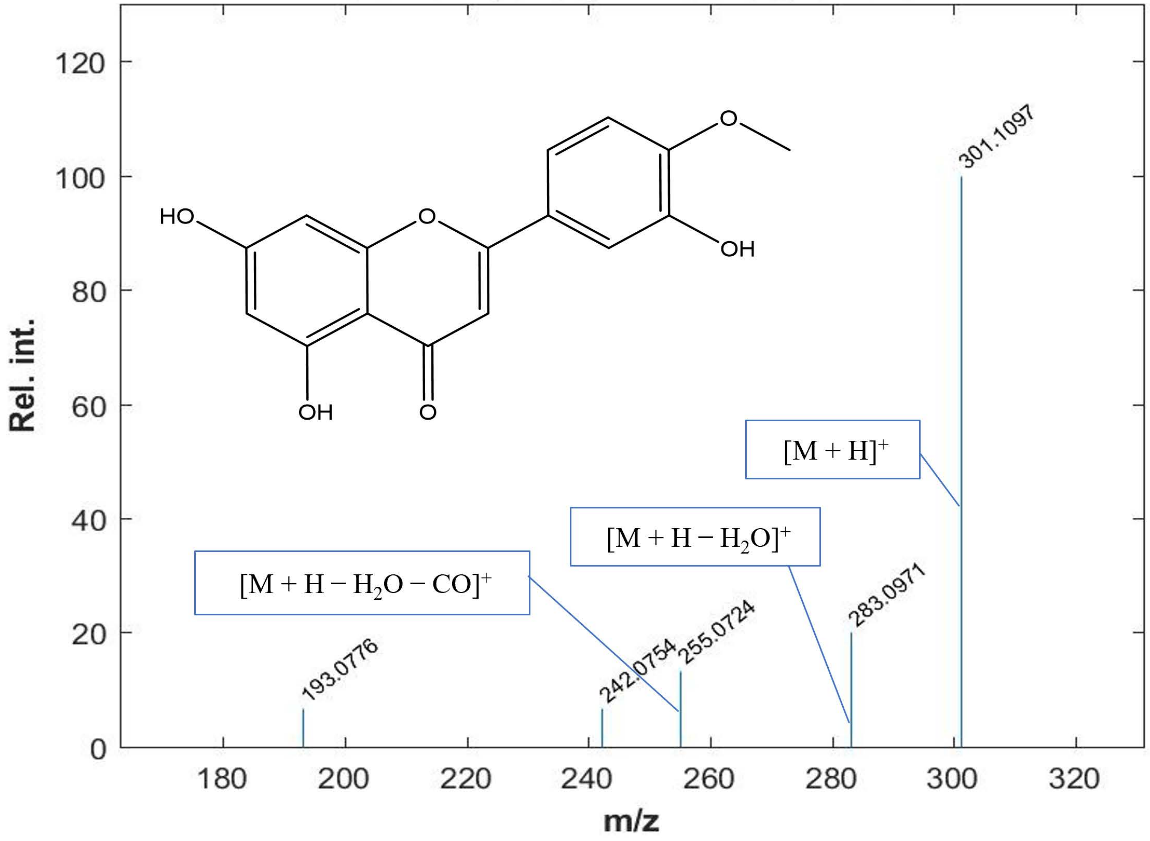

Figure S11. MS/MS spectrum of peak 46: 3, 5, 7-trihydroxy-4'-methoxyflavone (Diosmetin)

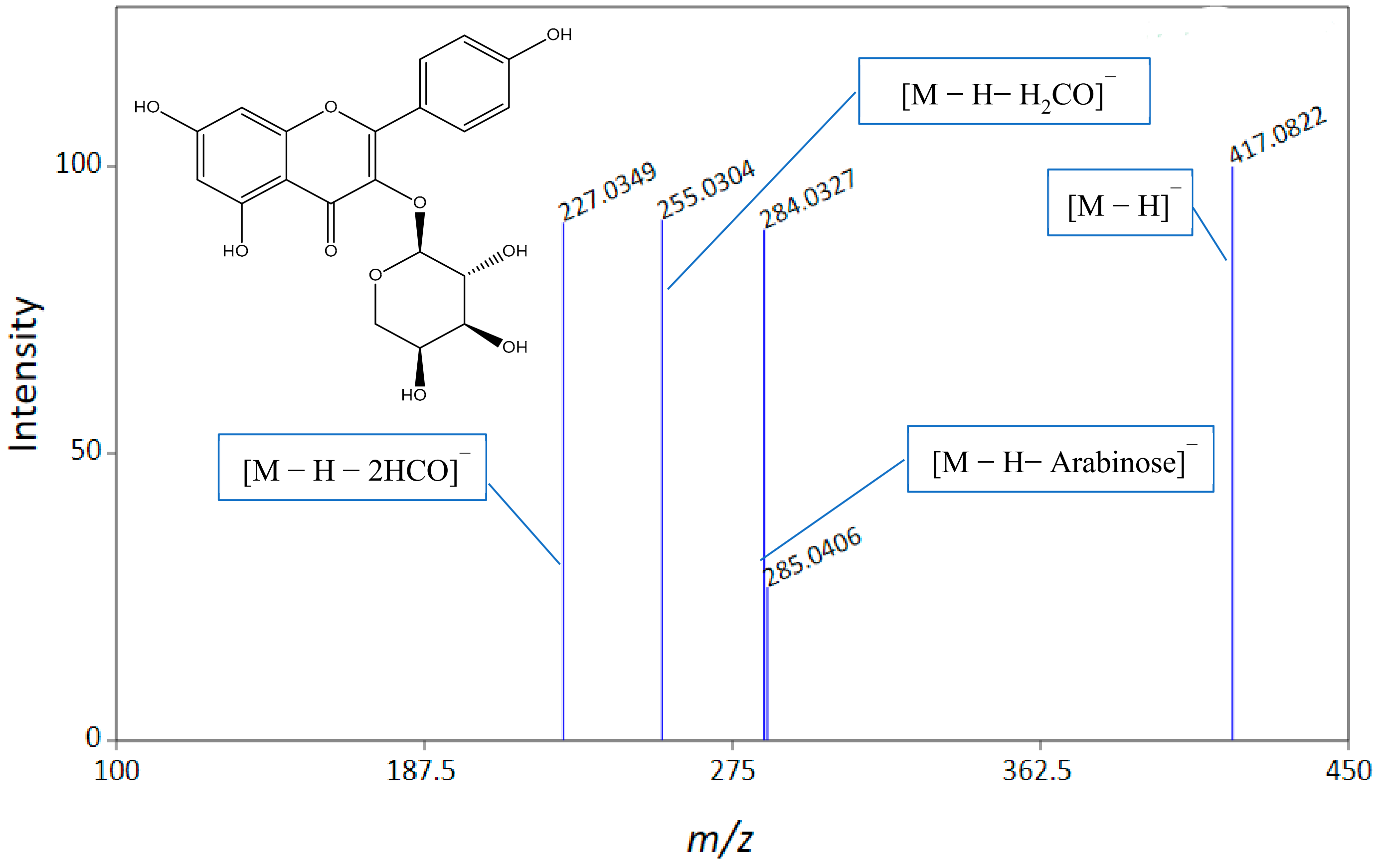

Figure S12. MS/MS spectrum of peak 12: Kaempferol-3-O-alpha-L-arabinoside

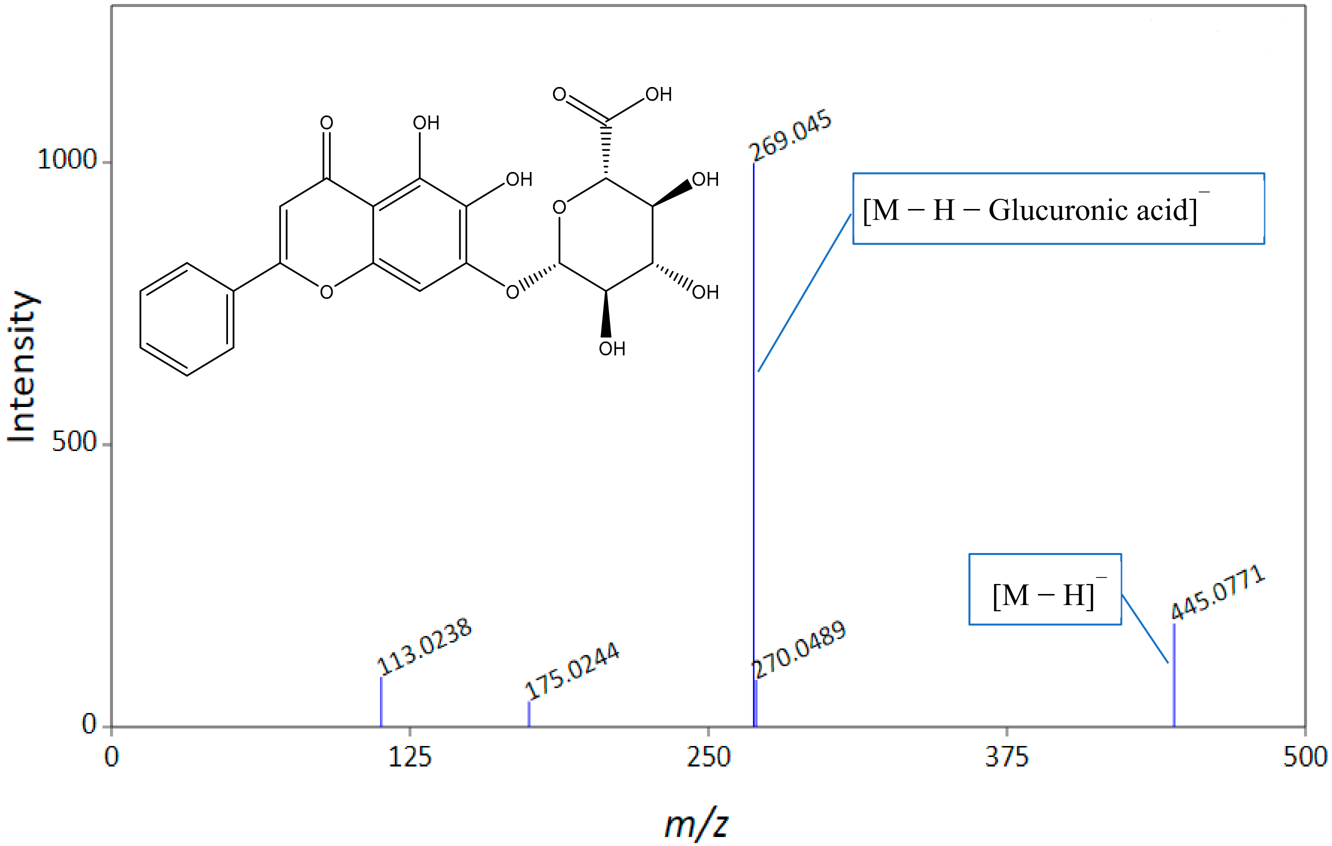

Figure S13. MS/MS spectrum of peak 35: Baicalein-7-*O*-glucuronide

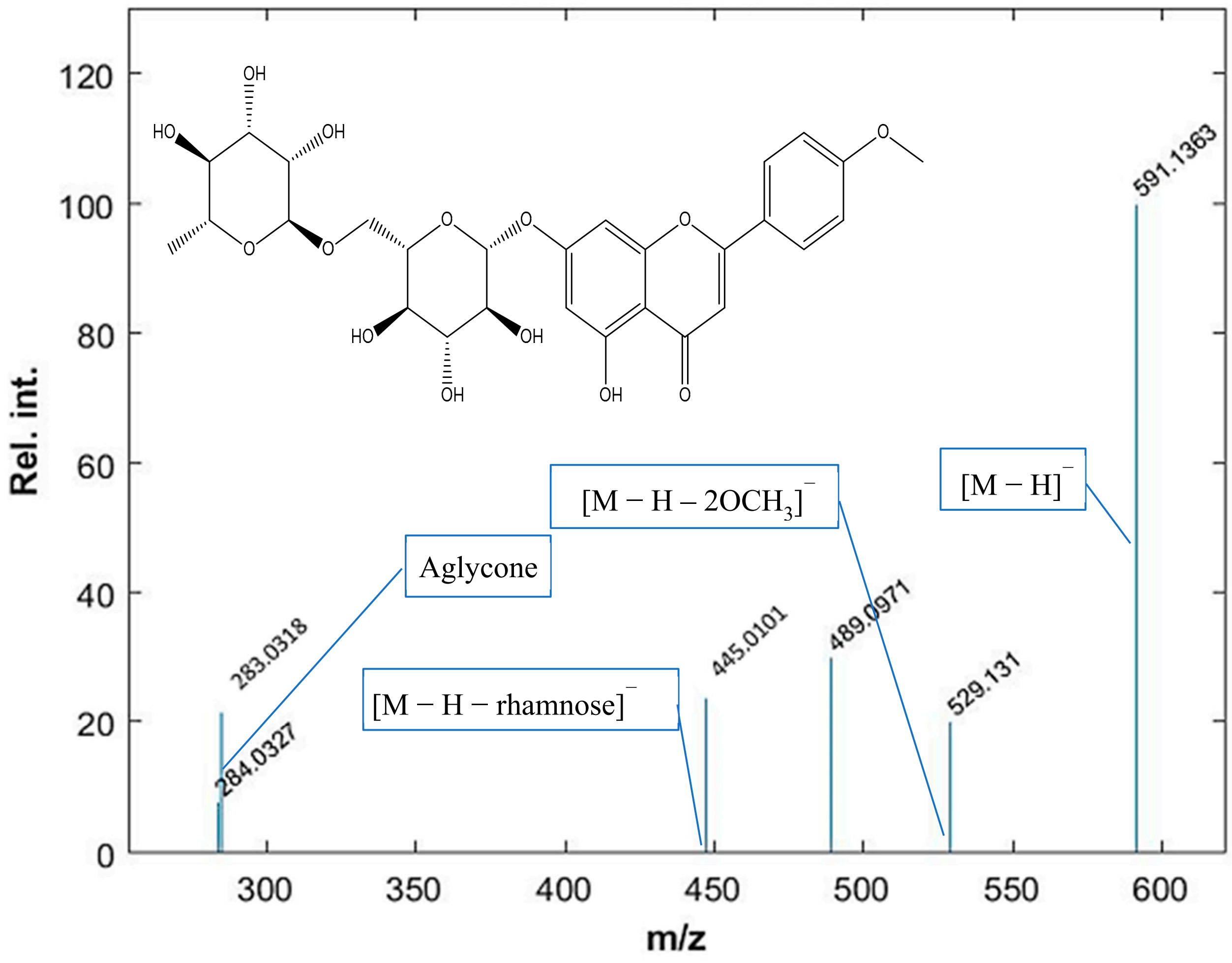

Figure S14. MS/MS spectrum of peak 38: Acacetin-*O*-rutinoside (linarin)

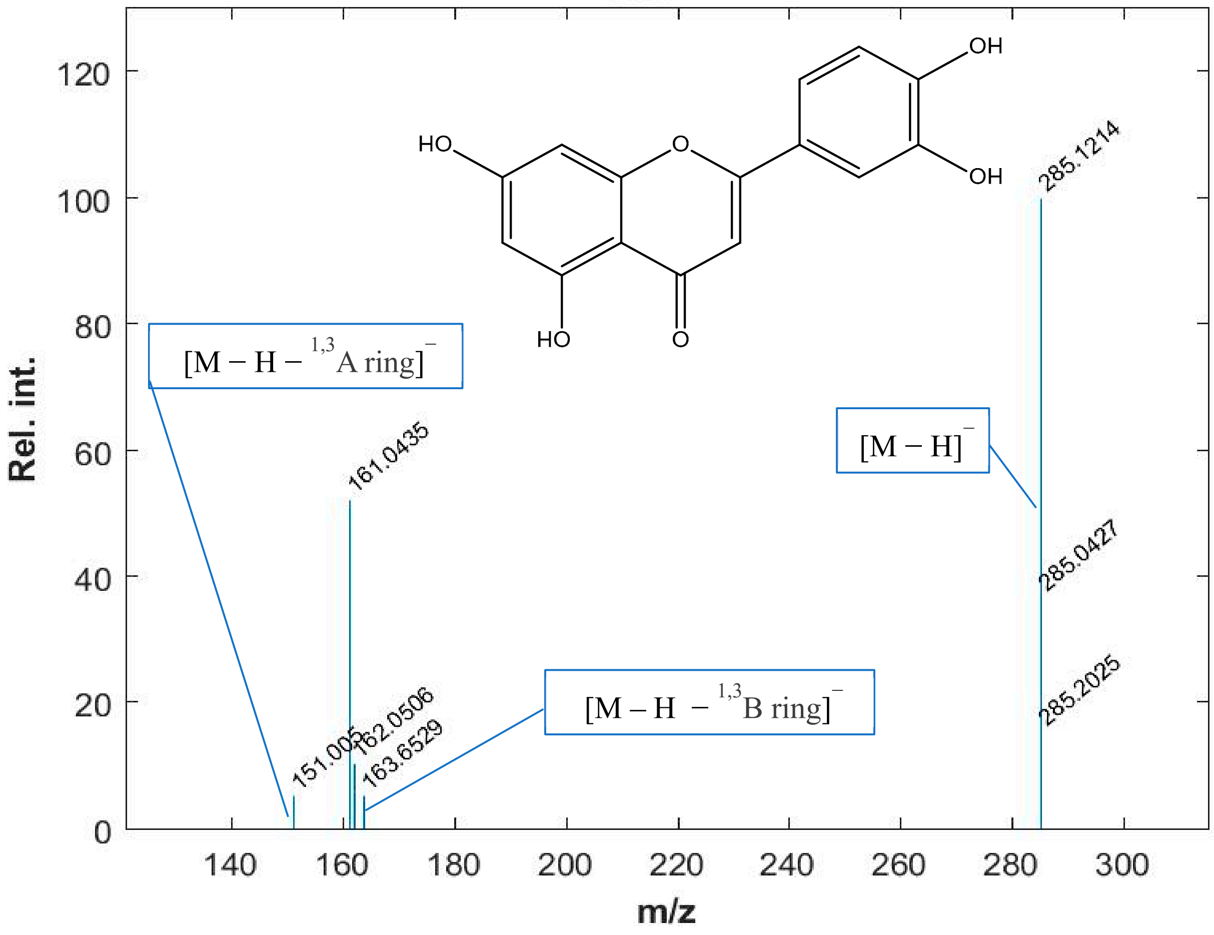

Figure S15. MS/MS spectrum of peak 39: Luteolin

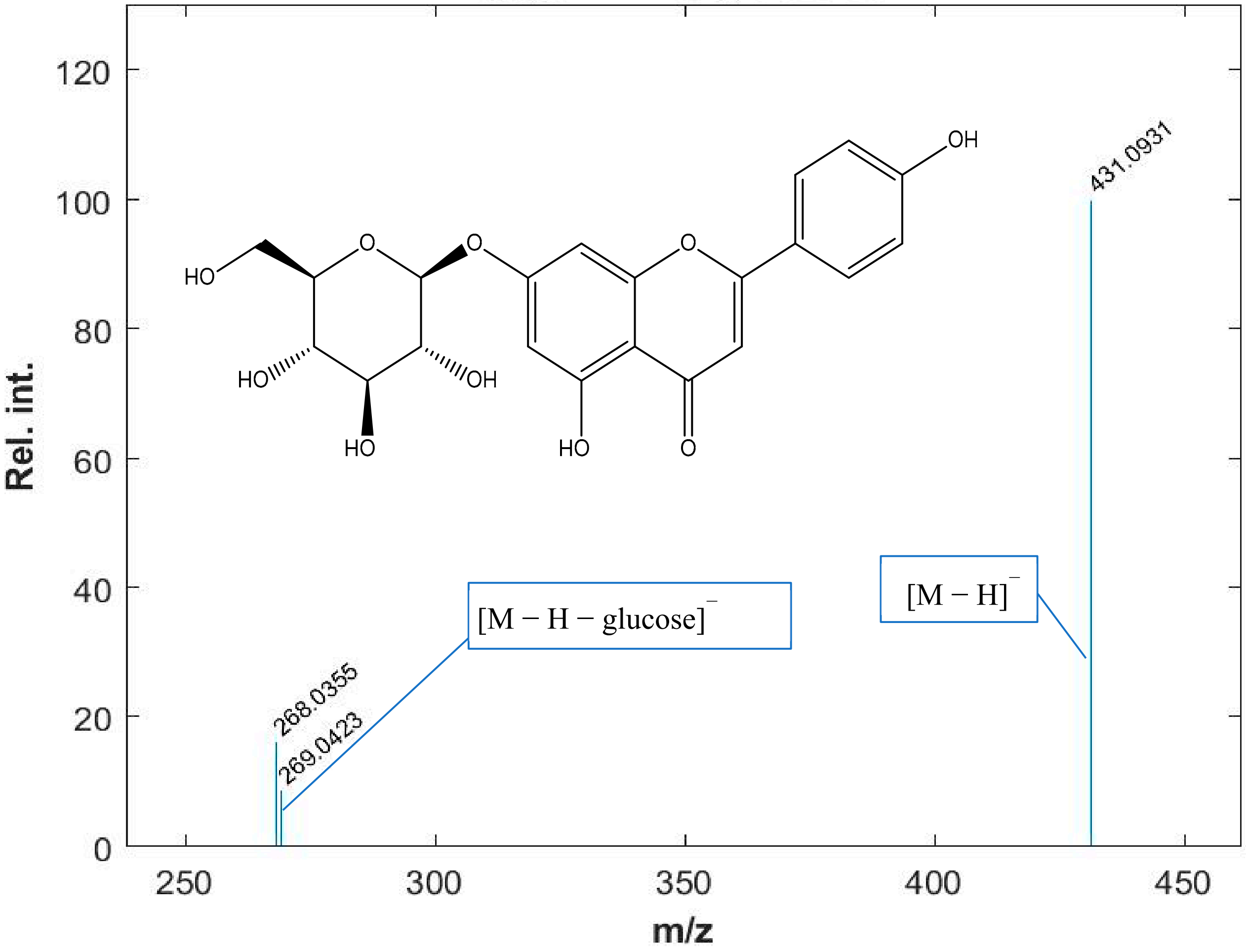

Figure S16. MS/MS spectrum of peak 47: apigenin-7-*O*-glucoside

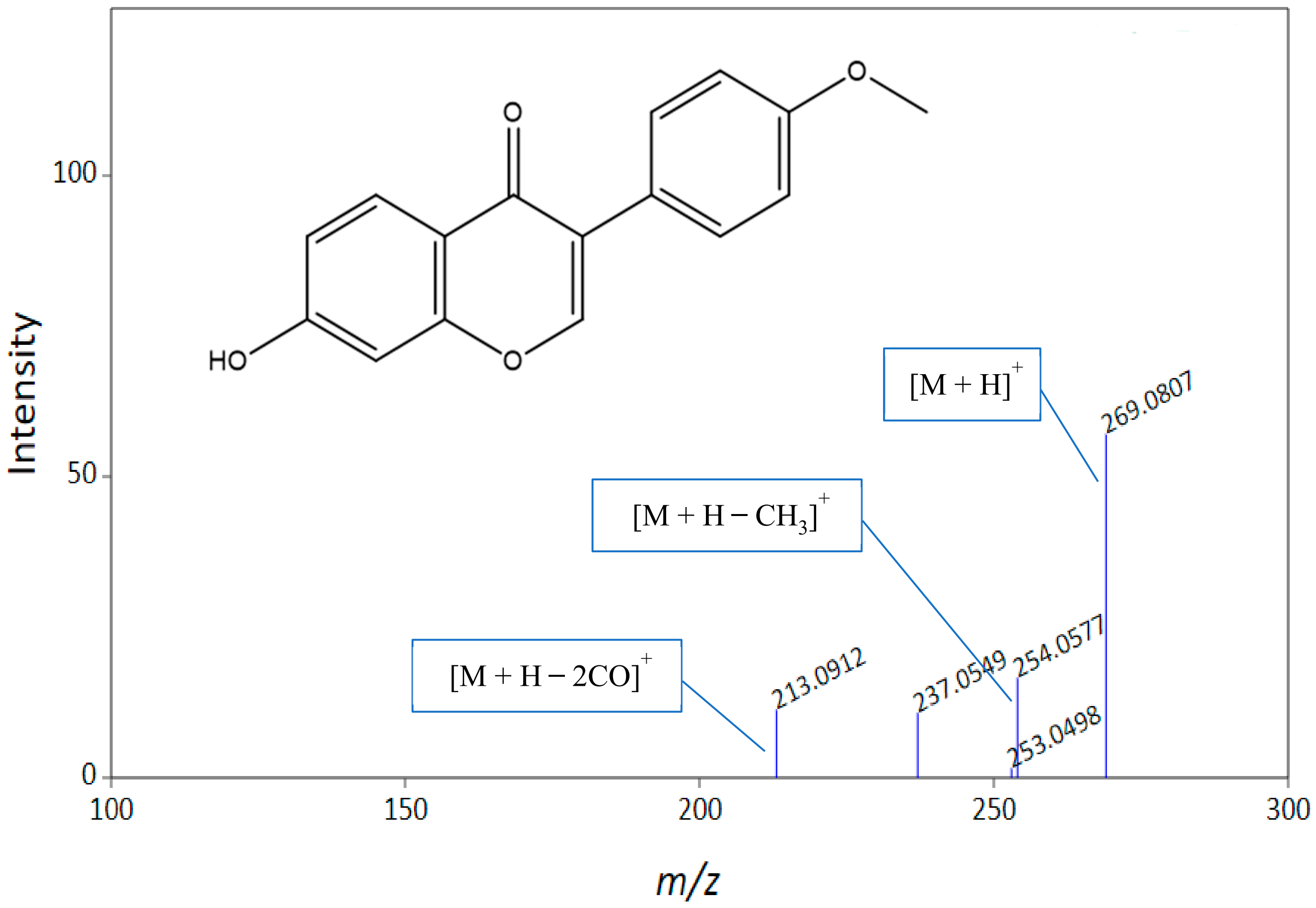

Figure S17. MS/MS spectrum of peak 51: Formononetin

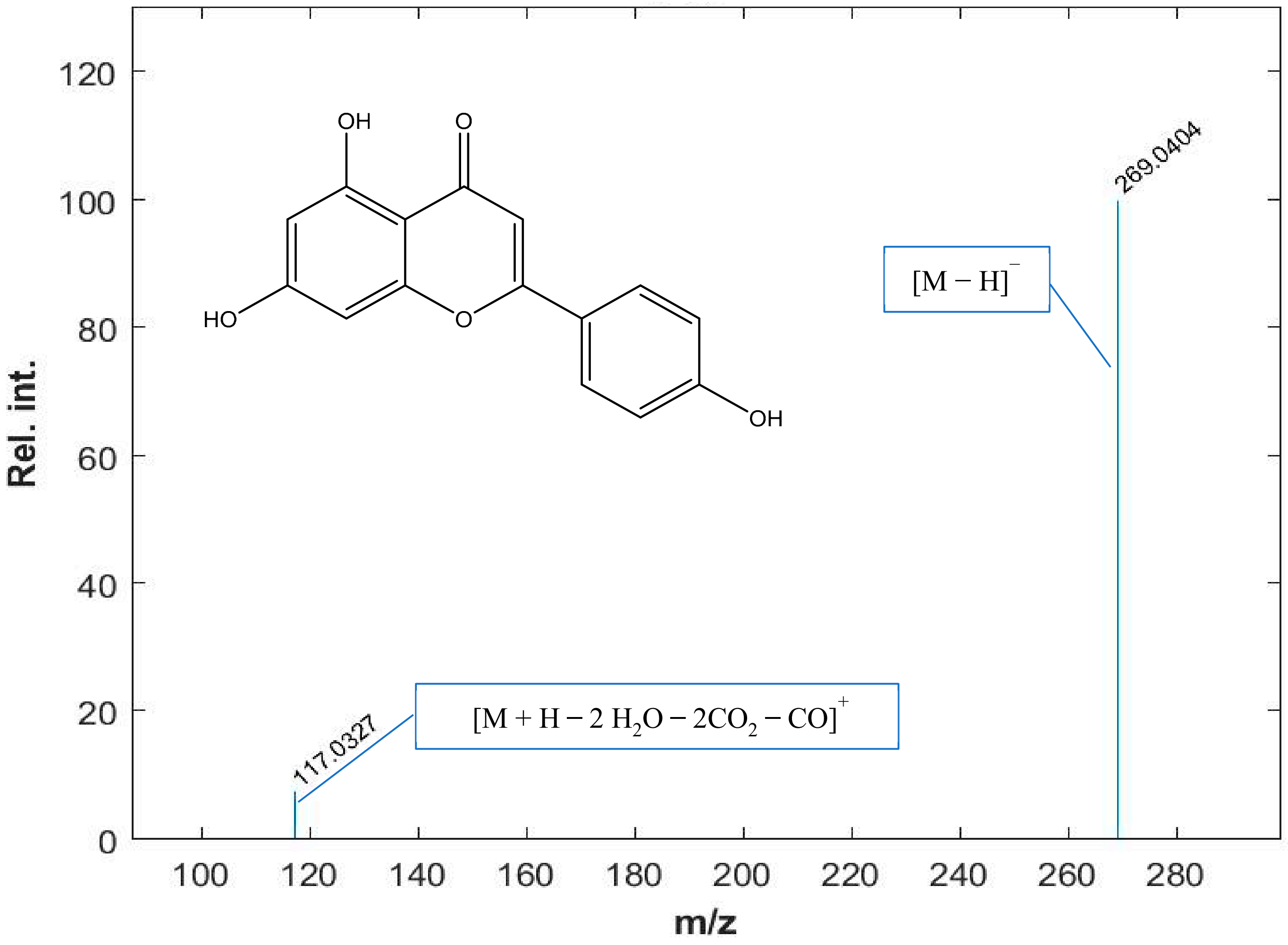

Figure S18. MS/MS spectrum of peak 39: Apigenin

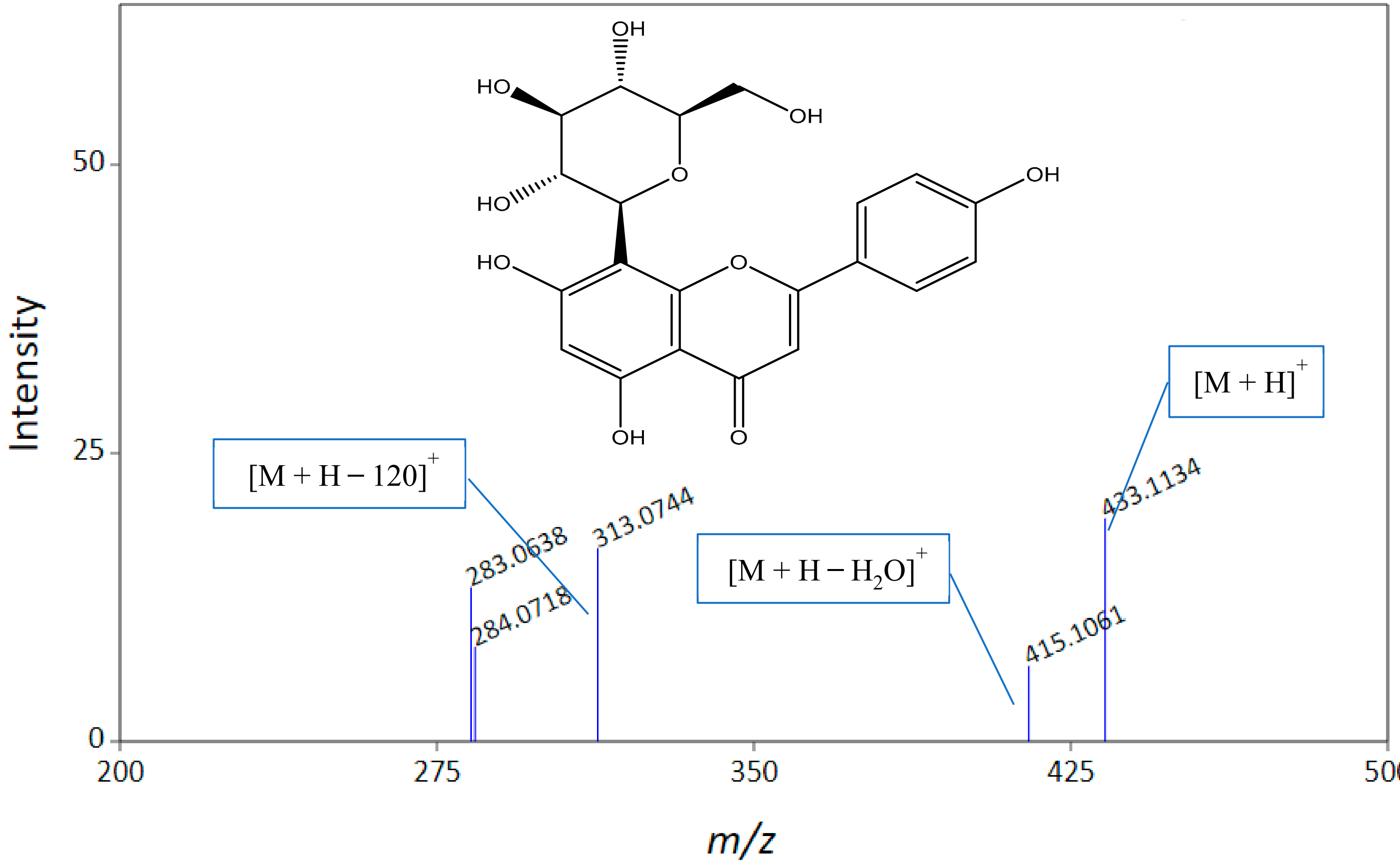

Figure S19. MS/MS spectrum of peak 54: Apigenin 8-C-glucoside (vitexin)

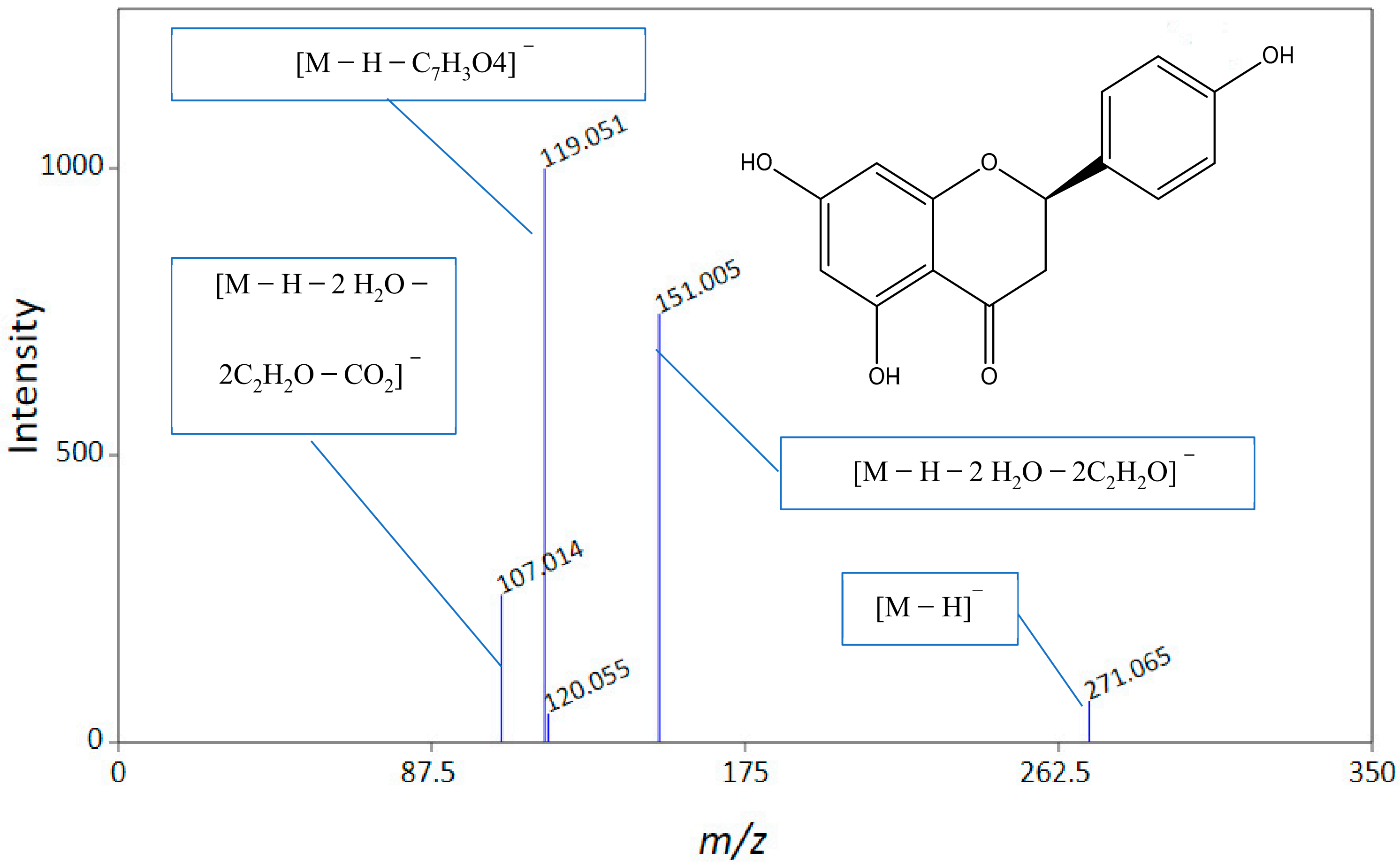

Figure S20. MS/MS spectrum of peak 1: Naringenin

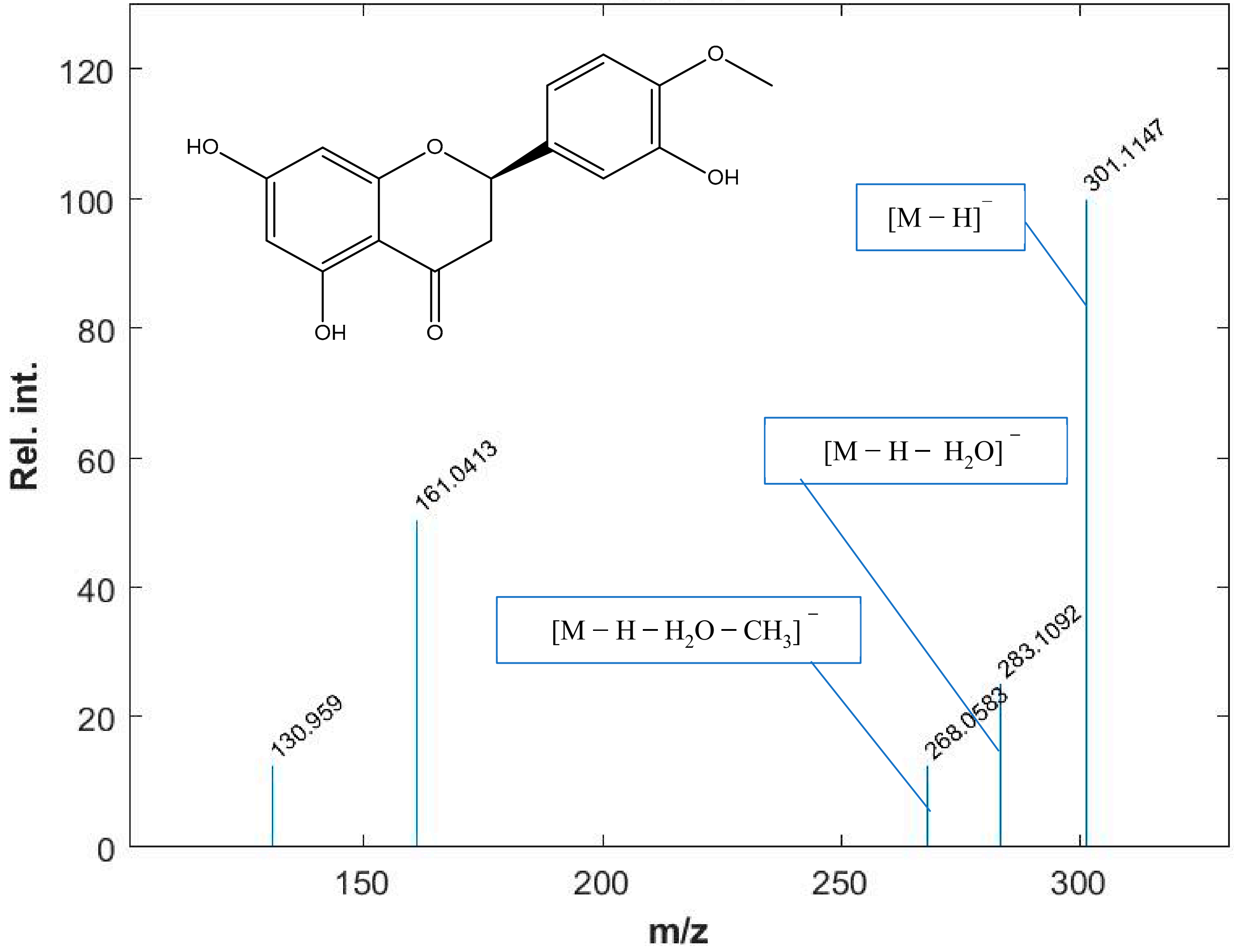

Figure S21. MS/MS spectrum of peak 45: Hesperetin

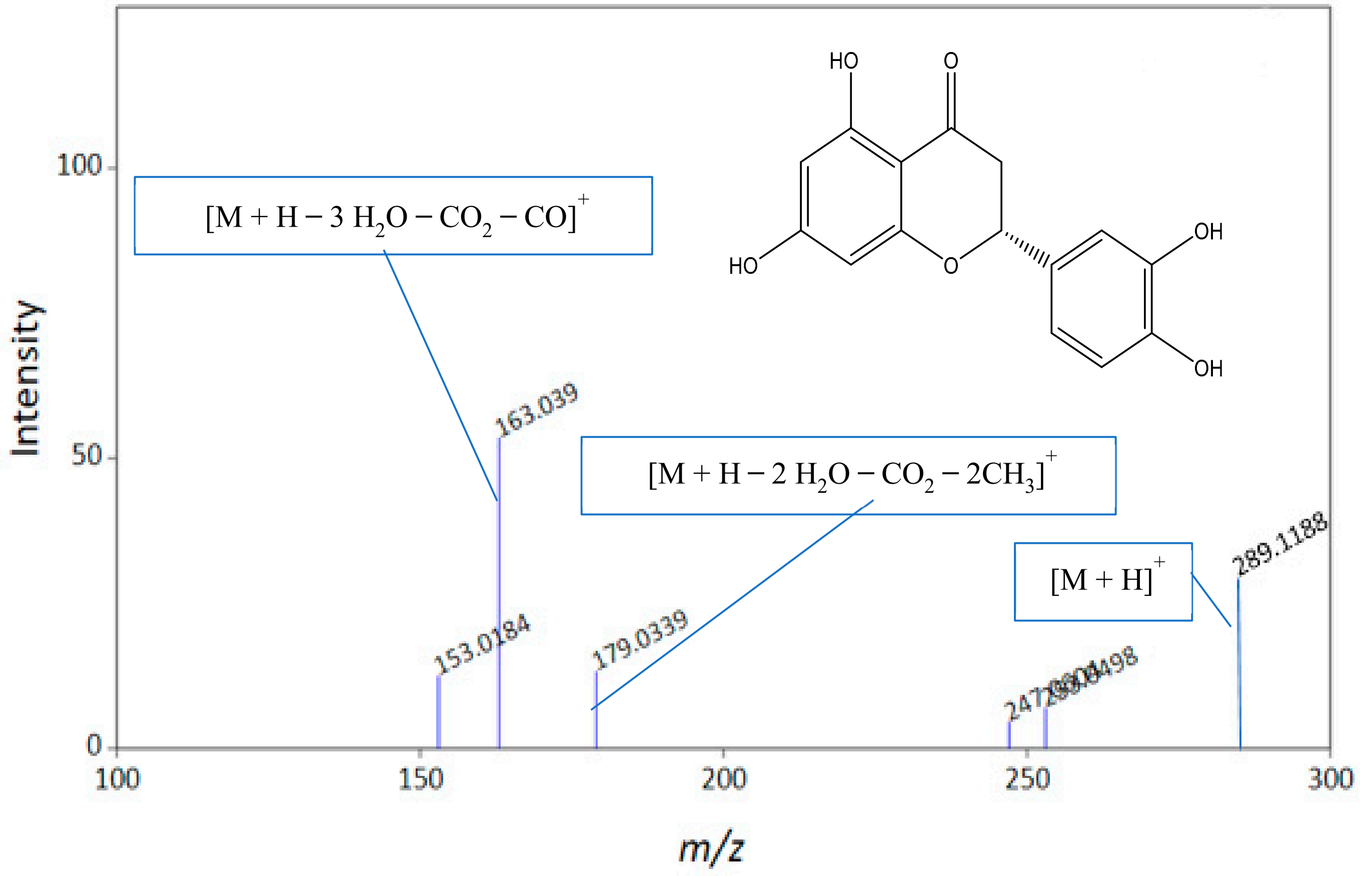

Figure S22. MS/MS spectrum of peak 53: 3' 4' 5 7-tetrahydroxyflavanone

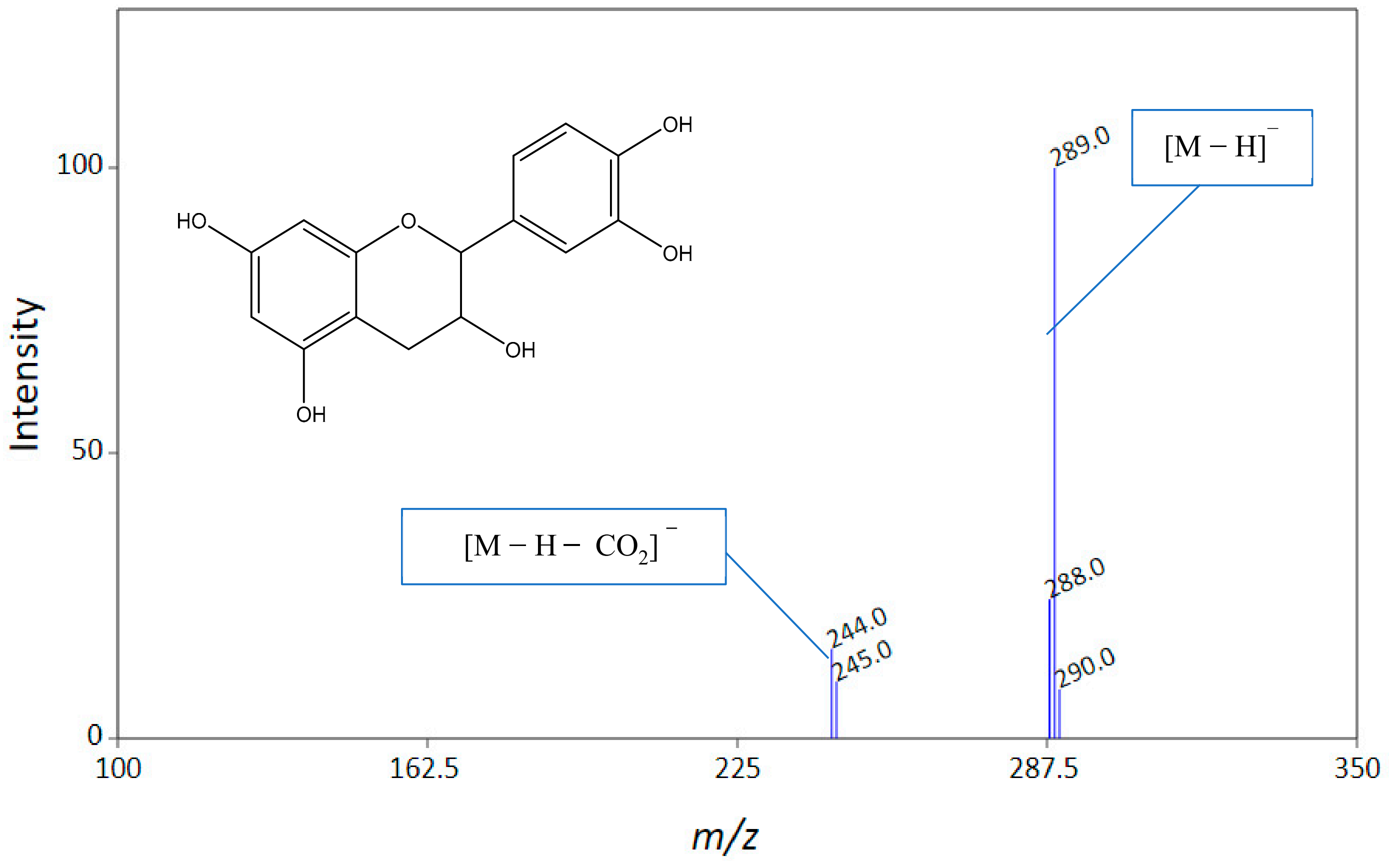

Figure S23. MS/MS spectrum of peak 34: Catechin

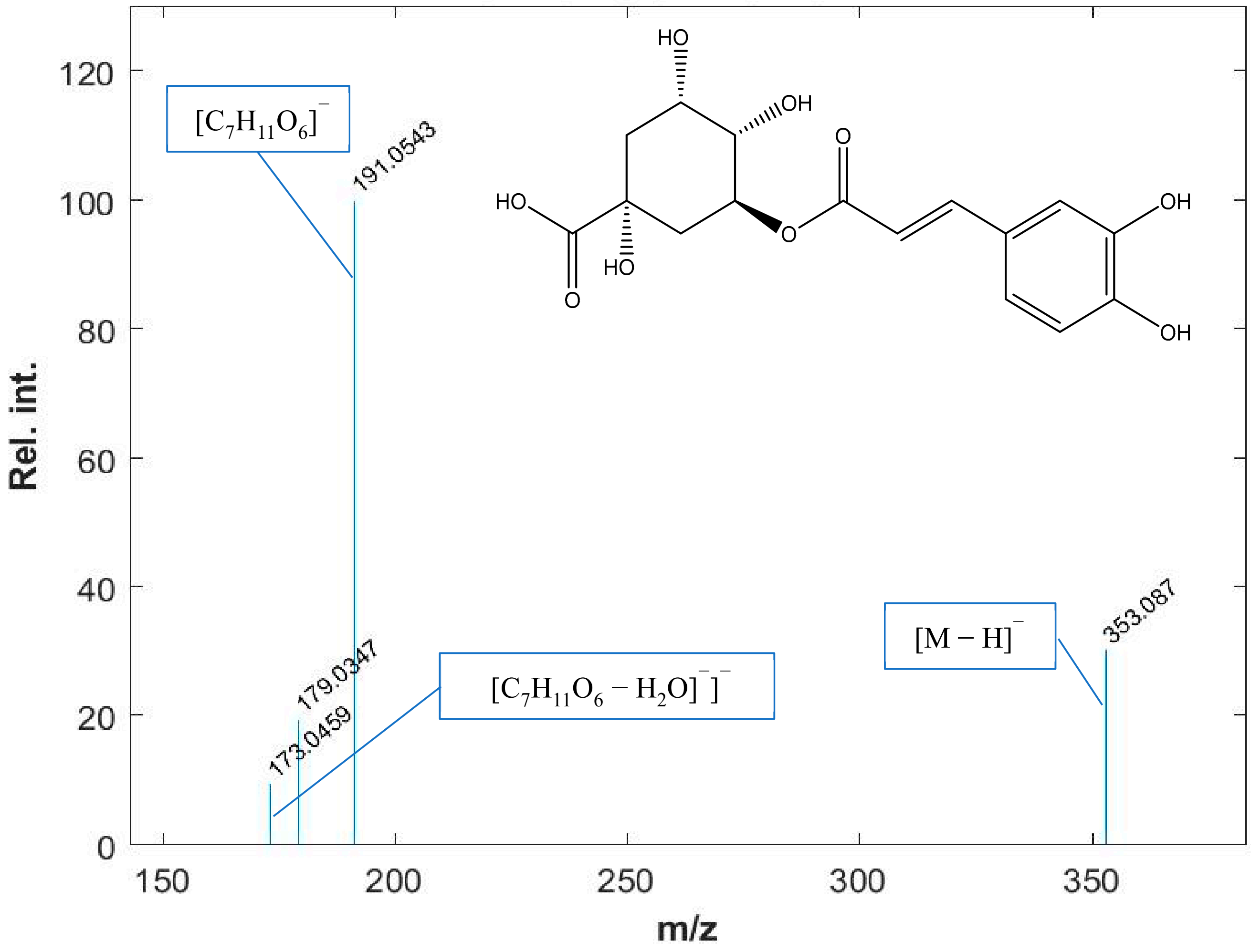

Figure S24. MS/MS spectrum of peak 19: Chlorogenic acid

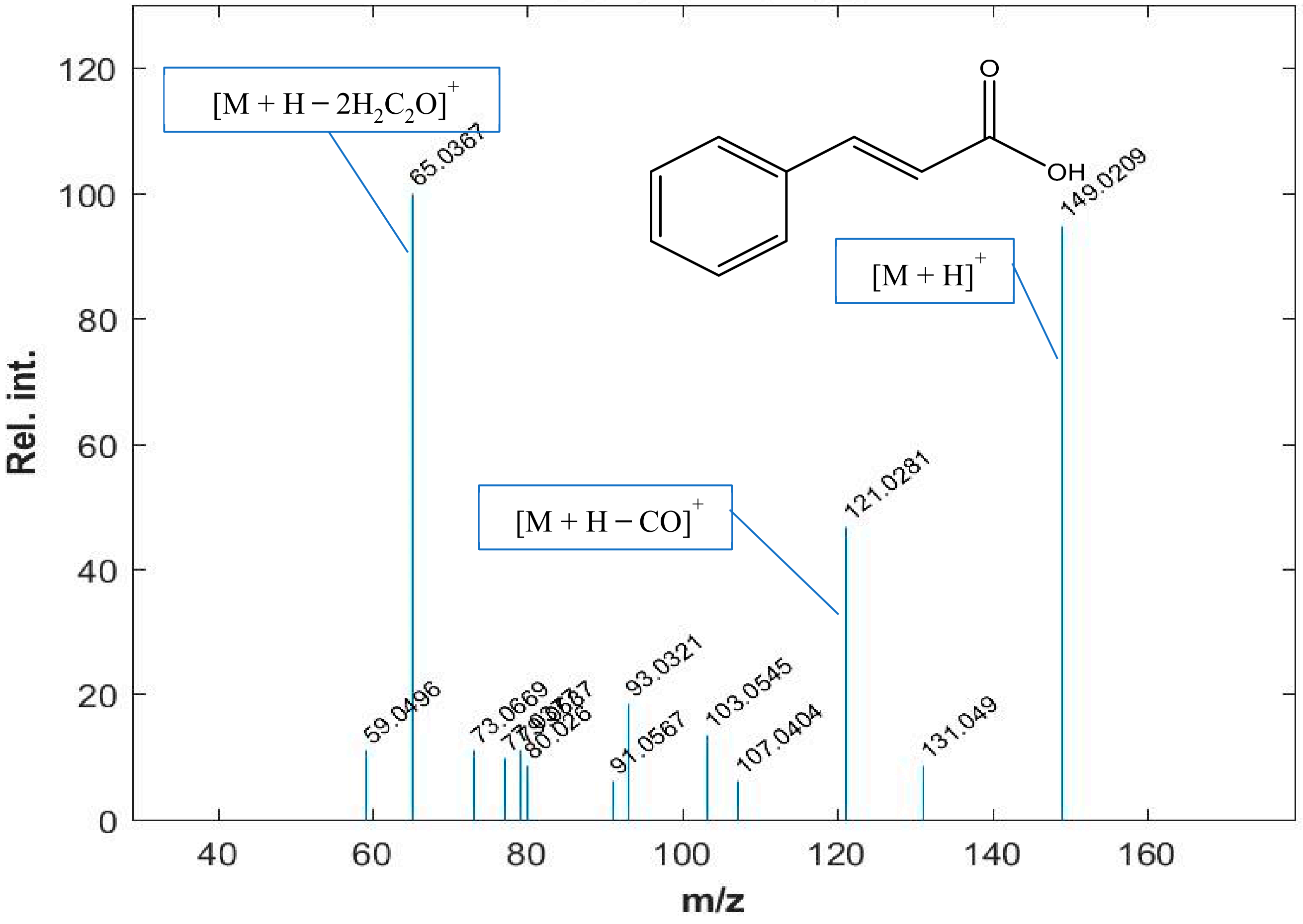

Figure S25. MS/MS spectrum of peak 24: *Trans*-Cinnamate

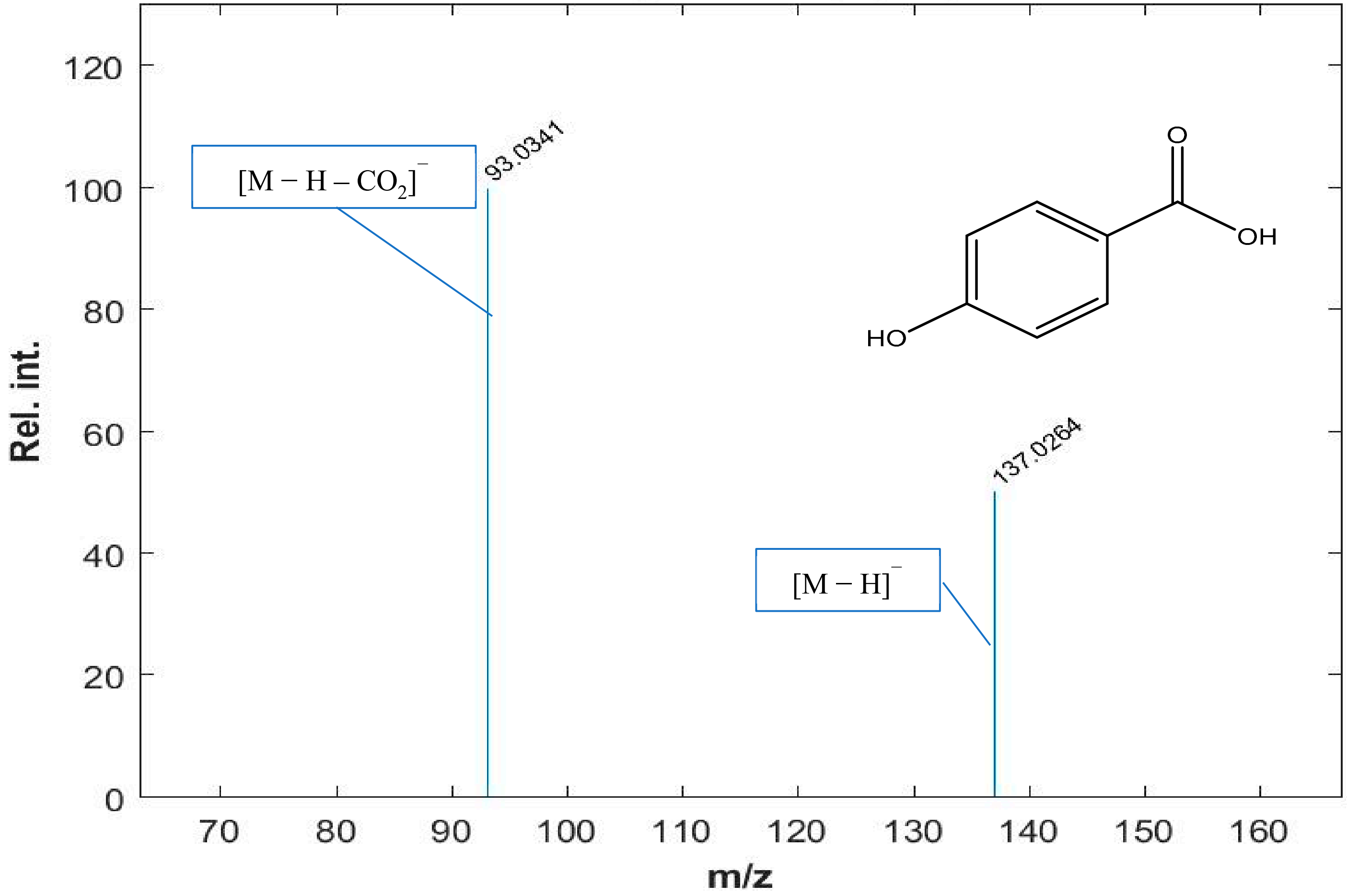

Figure S26. MS/MS spectrum of peak 26: *P*-Hydroxybenzoic acid

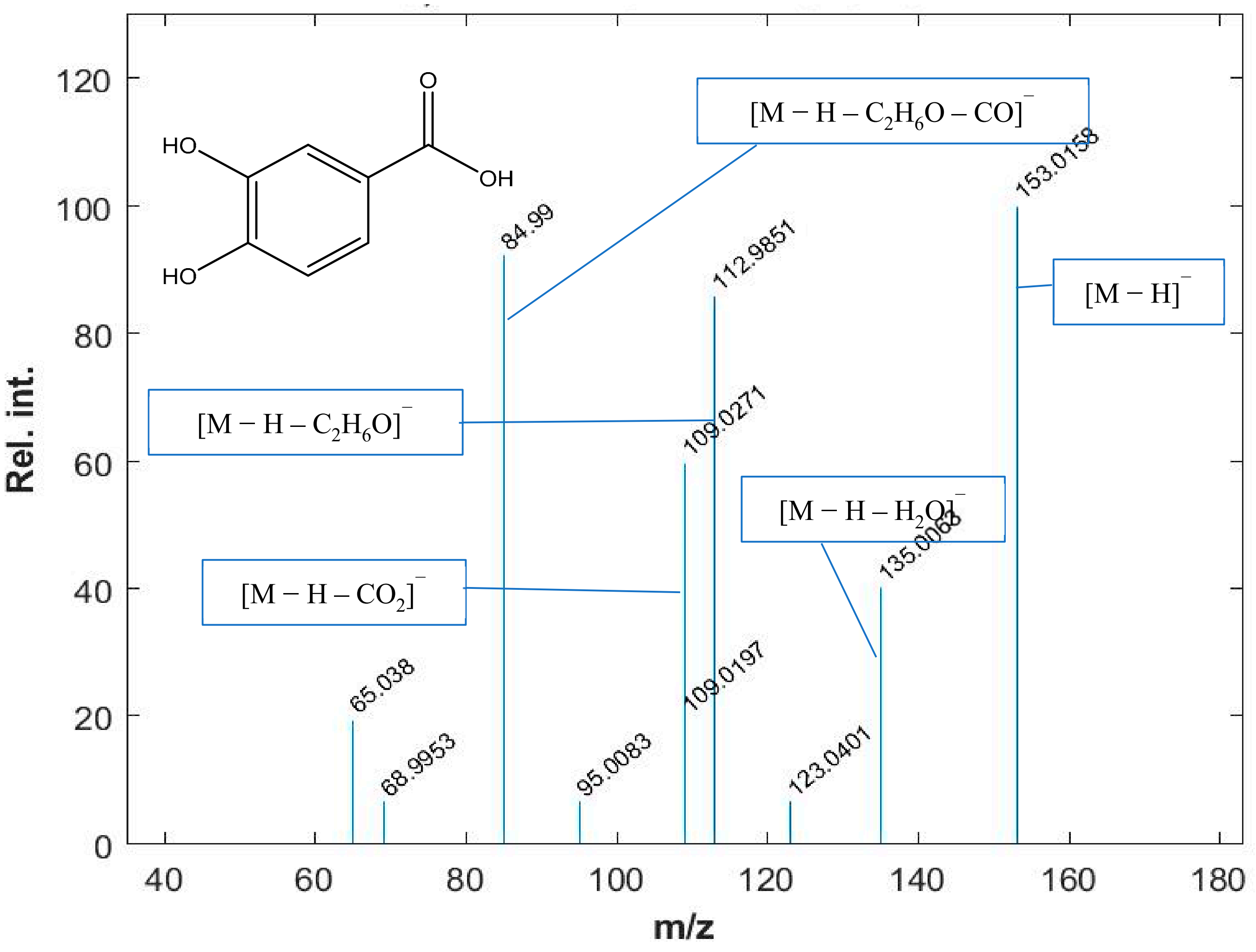

Figure S27. MS/MS spectrum of peak 31: 3,4-Dihydroxybenzoic acid (Protocatechuic acid)

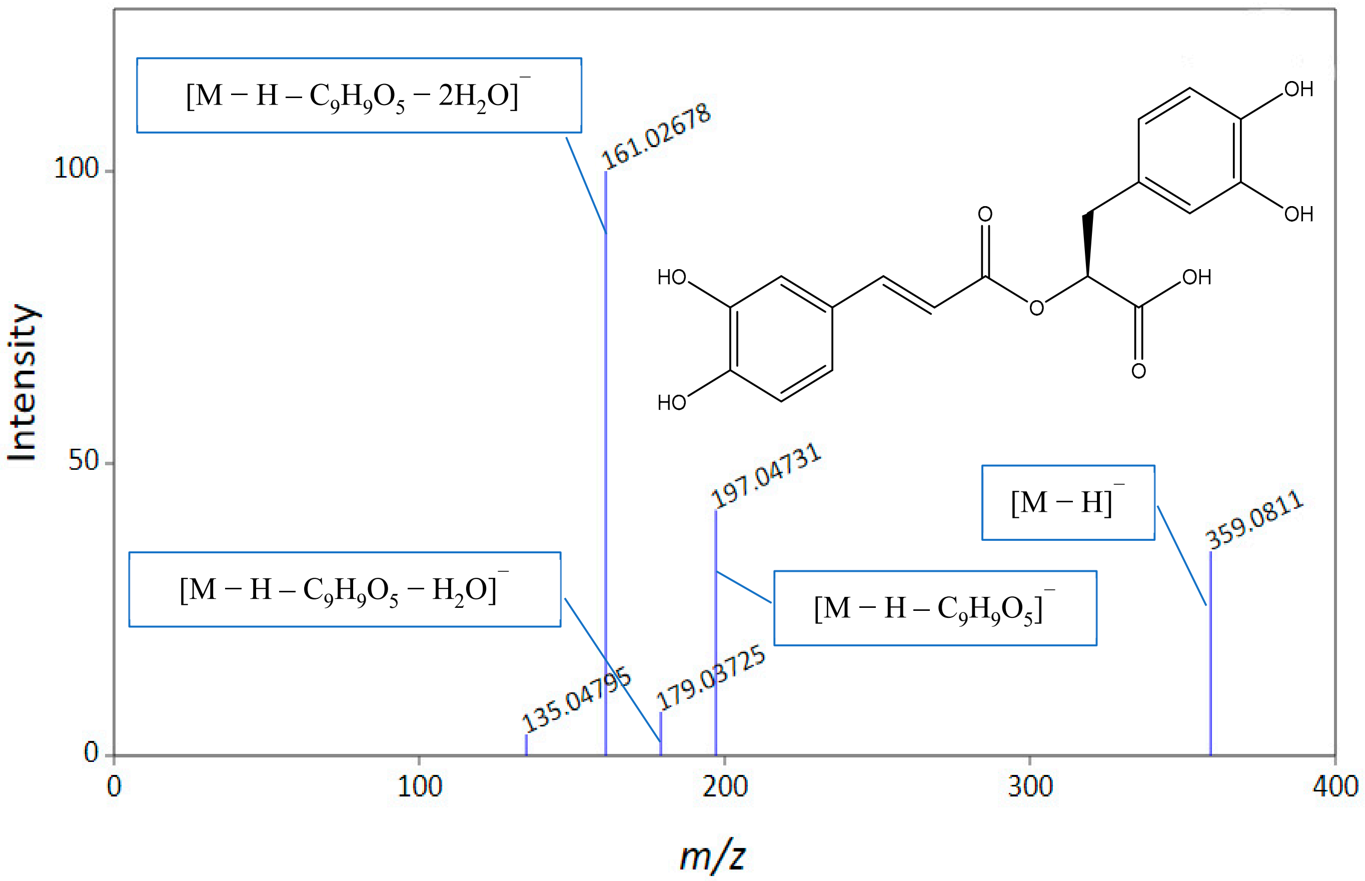

Figure S28. MS/MS spectrum of peak 48: Rosmarinic acid

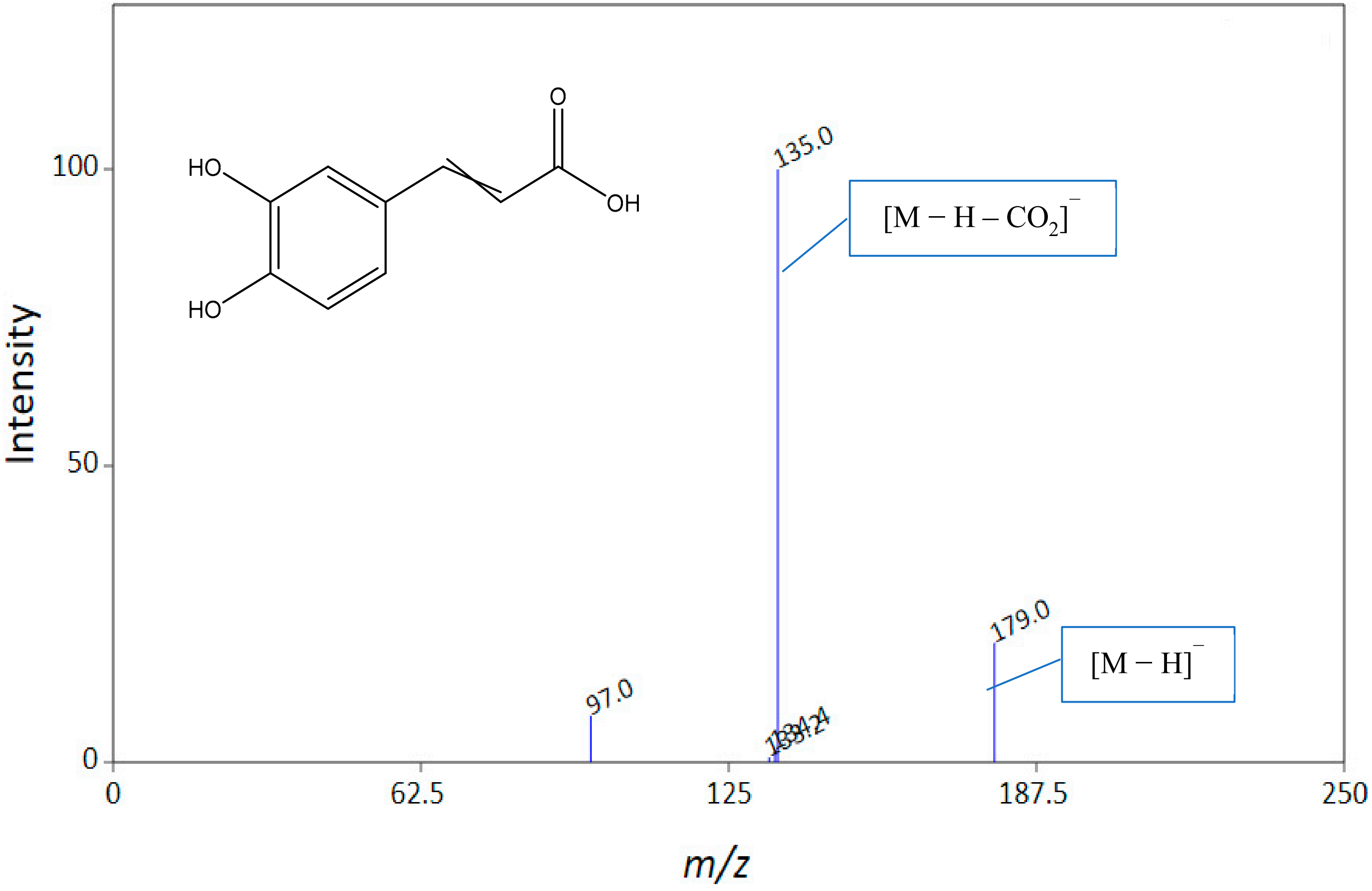

Figure S29. MS/MS spectrum of peak 55: Caffeic acid

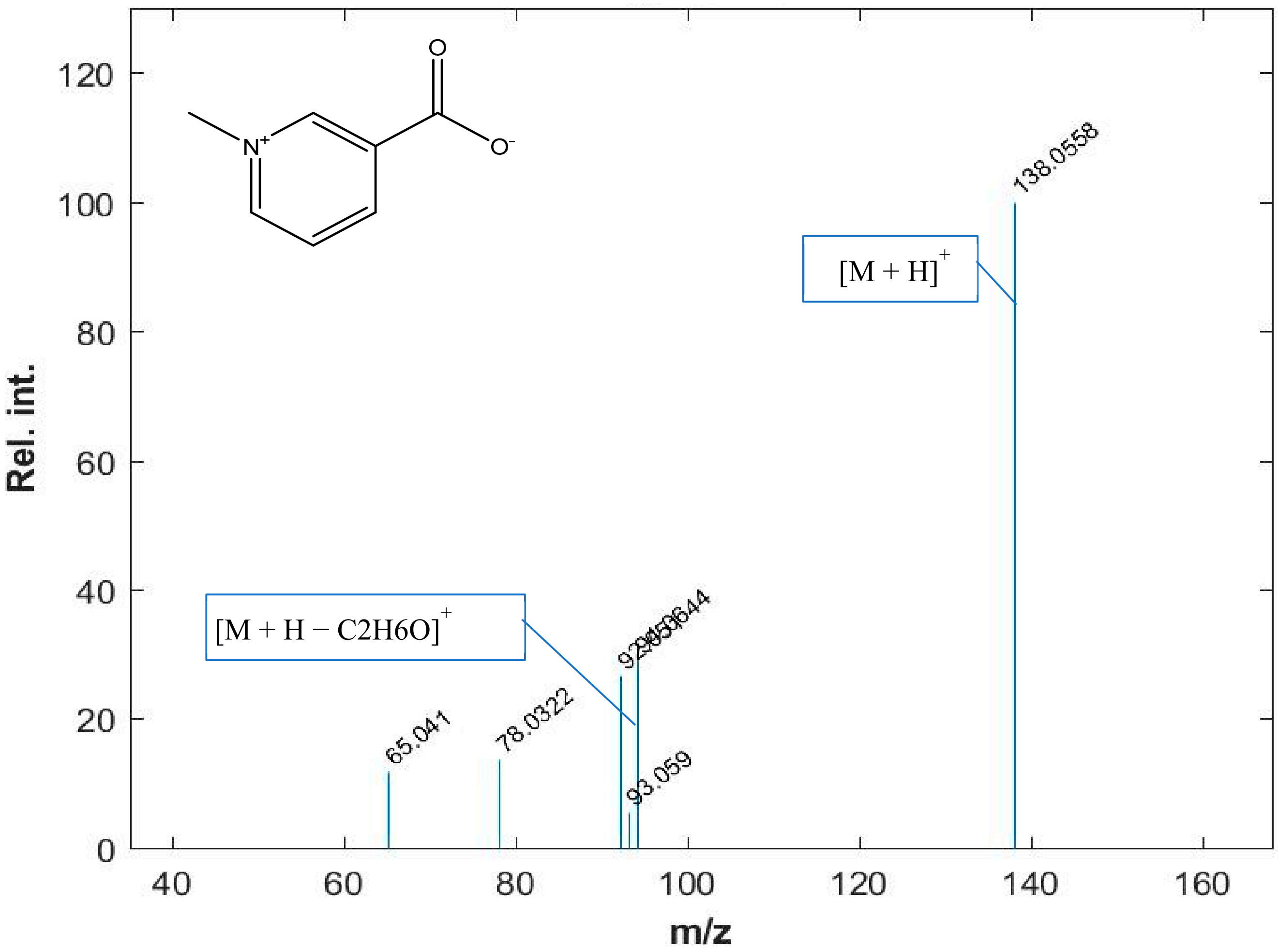

Figure S30. MS/MS spectrum of peak 11: Trigonelline

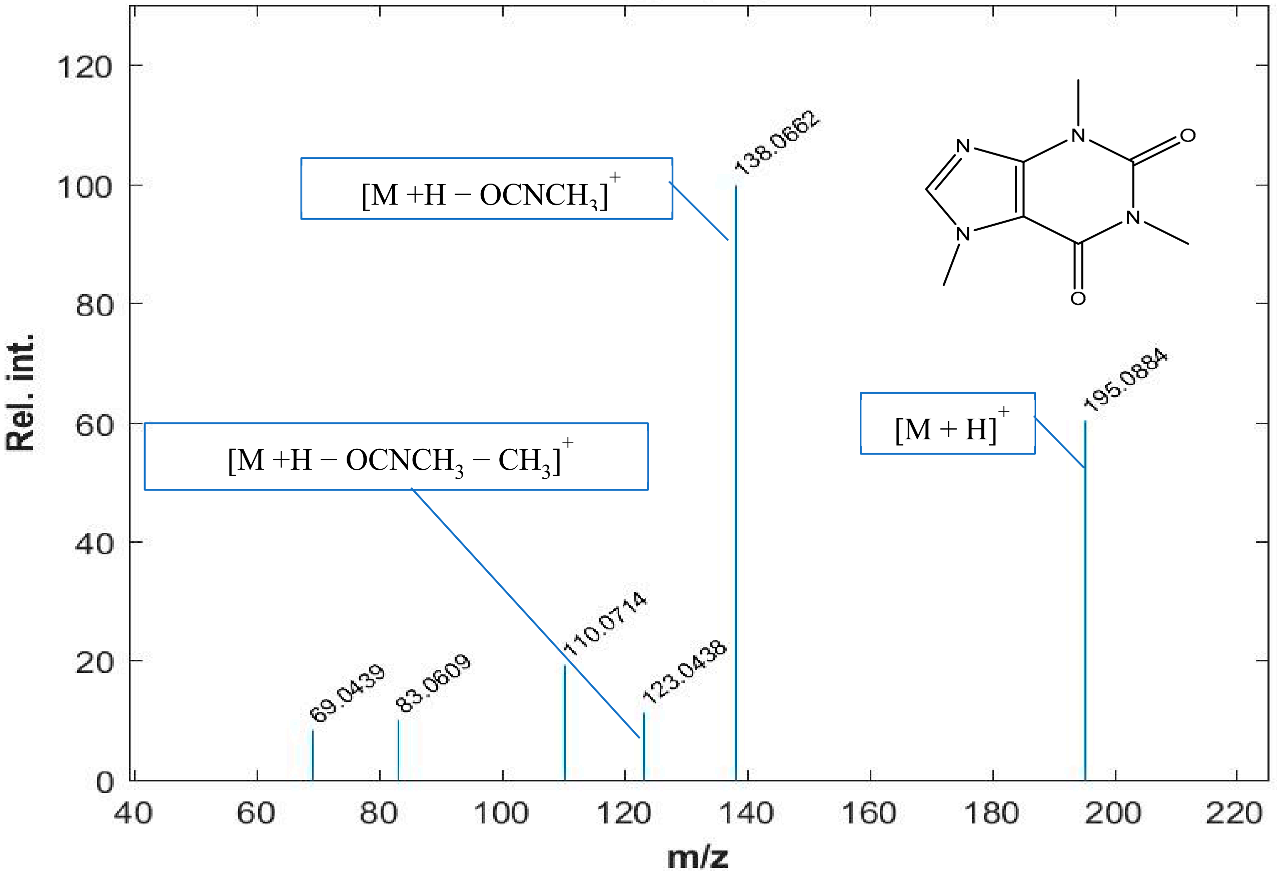

Figure S31. MS/MS spectrum of peak 32: Caffeine
